# Links between discrimination and cardiovascular health among socially stigmatized groups: A systematic review

**DOI:** 10.1371/journal.pone.0217623

**Published:** 2019-06-10

**Authors:** Gregory A. Panza, Rebecca M. Puhl, Beth A. Taylor, Amanda L. Zaleski, Jill Livingston, Linda S. Pescatello

**Affiliations:** 1 Department of Kinesiology, University of Connecticut, Storrs, CT, United States of America; 2 Department of Cardiology, Hartford Hospital, Hartford, CT, United States of America; 3 Rudd Center for Food Policy & Obesity, University of Connecticut, Hartford, CT, United States of America; 4 Department of Human Development & Family Studies, University of Connecticut, Storrs, CT, United States of America; 5 Department of Research Services, University of Connecticut, Storrs, CT, United States of America; University of Calgary, CANADA

## Abstract

**Background:**

There is a high prevalence of cardiovascular disease across diverse groups in the U.S. population, and increasing research has identified stigma as a potential barrier to cardiovascular disease prevention and treatment. This systematic review examines evidence linking discrimination and cardiovascular health among socially stigmatized groups.

**Study Design:**

Six databases were systematically reviewed from inception through February 2018 for studies with adult subjects, focusing on cardiovascular health indicators among social groups stigmatized because of their gender, race/ethnicity, age, body weight/obesity, or sexual orientation. The Newcastle-Ottawa Scale was used to evaluate the methodological quality and risk of bias for nonrandomized studies, and the Cochrane Collaboration 7-item domain for randomized controlled and experimental trials.

**Results:**

The search identified 84 eligible studies published between 1984 and 2017. Studies retrieved were categorized according to demonstrated links between stigma and cardiovascular disease risk factors including blood pressure (*n* = 45), heart rate variability (*n* = 6), blood/saliva cardiovascular biomarkers (*n* = 18), as well as other indicators of cardiovascular health (*n* = 15). Based on the findings from included studies, 86% concluded that there was a significant relationship among stigma or discrimination and cardiovascular health indicators among socially stigmatized groups. However, there were varying degrees of evidence supporting these relationships, depending on the type of discrimination and cardiovascular health indicator. The current evidence implies an association between perceived discrimination and cardiovascular health. However, a majority of these studies are cross-sectional (73%) and focus on racial discrimination (79%), while using a wide variety of measurements to assess social discrimination and cardiovascular health.

**Conclusions:**

Future research should include longitudinal and randomized controlled trial designs, with larger and more diverse samples of individuals with stigmatized identities, using consistent measurement approaches to assess social discrimination and its relationship with cardiovascular health.

## Introduction

Many American adults face social stigmatization, the experience of being discredited and/or rejected because of a particular characteristic or attribute that is deemed socially undesirable [1]. Societal stigma can lead to prejudice, stereotyping, unfair treatment, discrimination, and remains common among a number of groups in Western society. According to the National Survey of Midlife Development in the United States, gender was the most commonly reported type of discrimination in America from 1995/6 through 2005/6, particularly among women (27%) [2]. Other reported types of discrimination included race (17% men, 9% women), age (10% men, 11% women), weight [5% men, 10% women; 40% for adults with a body mass index (BMI) ≥35kg/m^2^] [3], other aspects of physical appearance (8% men, 4% women), and ethnicity/nationality (6% men, 3% women) [2]. Recent surveys continue to show a high prevalence of discrimination amongst these socially stigmatized groups [4].

The Centers for Disease Control and Prevention (CDC; [5] and the World Health Organization (WHO; [6] recognize societal stigma as a public health priority because of its adverse effects on effective prevention and treatment of diseases and its potential to accelerate disease processes. As a result, the WHO adopted Goal 16 of the 2030 Agenda for Sustainable Development which encourages inclusive societies that promote non-discrimination [6]. This initiative aims to counter negative consequences of stigma including suffering, delayed treatment, declines in daily activities, and unfair access to health insurance and appropriate medical care, [5] all of which make stigmatized populations more susceptible to chronic disease and mortality [7,8]-[9].

Recent research suggests that acute and chronic exposure to societal stigma and discrimination is associated with an increase in a variety of adverse cardiovascular health outcomes [10]. Underlying mechanisms responsible for this association may be attributable to the way the body responds to the emotional distress of stigma and discrimination as a stressor. There are several well-known acute physiological changes that occur when the body responds to a stressor [11], known as the ‘fight or flight’ response. Acute stress (i.e., stress that is momentary or short-term) can cause an increase in heart rate and blood pressure, and a secretion of stress hormones (e.g., adrenaline, noradrenaline, and cortisol) [11]. When acute stressors occur over time they become chronic stress and can have significant health implications on the cardiovascular system due to chronic sympathetic nervous system stimulation [11], ultimately affecting cardiovascular disease processes. Chronic stress can cause heart rate and blood pressure to remain elevated, while vasoconstriction can occur if endothelial dysfunction is present, leading to myocardial ischemia. Atherosclerosis can also develop due to endothelial dysfunction and injury as well as arrhythmias due to an increase in pro-arrhythmogenic potential. Furthermore, there is an increased risk for thrombosis due to platelet activation, hemostatic changes, and hemocentration [11].

This evidence supporting the association between societal discrimination and an increase in adverse cardiovascular health outcomes has been documented across several types of stigmatization including race [10], weight [12], gender [13], and sexual orientation [14], and across different indices of cardiovascular health such as blood pressure (BP) [13], heart rate (HR)/heart rate variability (HRV) [15], and cardiovascular biomarkers (e.g., cortisol) [16]. Since CVD is the leading cause of death in the U.S. and world [17], it is imperative to better understand the role that societal discrimination plays in cardiovascular health among socially stigmatized groups. Previous reviews have demonstrated a link among perceived racial discrimination and BP, [10,18] however, these reviews have primarily focused on racial discrimination and hypertensive status. To the best of our knowledge, our review is the first to examine multiple indices of cardiovascular health among several socially stigmatized groups. A systematic review such as this is needed to obtain a better understanding of the evidence, gaps in knowledge, and key questions that can inform and advance research on this important topic. Therefore, the aim of this review is to: a) provide an overview of the scientific evidence linking discrimination and indicators of cardiovascular health among socially stigmatized groups; b) compare research findings of cardiovascular health indicators across stigmatized groups; c) summarize the strengths and limitations of the current literature; and d) identify future directions to advance this field of study.

## Methods

### Search protocol

Our systematic review followed the standards of the Preferred Reporting Items for Systematic Reviews and Meta-Analyses (PRISMA) Statement [19]. Boolean searches in PubMed, PsycINFO, CINAHL, Sociological Abstracts, Academic Search Premier, and Scopus databases identified studies assessing the relationship between discrimination and cardiovascular health among commonly stigmatized groups. We searched databases from their inception through February 2018. The search was guided by three themes including stigma, socially stigmatized groups, and cardiovascular health. Details regarding the search strategy, including a full list of keywords, are available in S1 File. Two authors (GAP and ALZ) independently extracted and entered study information with high reliability across categorical (mean Cohen *κ* = 0.92) and continuous (mean Pearson *r* = 0.94) variables [20]. All disagreements were resolved by consensus. Data extracted included study author, study design, study population, measure of stigma and/or discrimination, measure of cardiovascular health indices, length of study, and key study findings (S2 Table). We conducted separate searches for four cardiovascular health indicator categories including: 1) BP, because elevated BP or hypertension is the most prevalent, modifiable, and costly risk factor for CVD; [17] 2) HRV/HR, because reduced HRV has been shown to predict the increased risk of cardiac events [21] and previous studies have shown the association between increased HR and increased risk of CVD [11]; 3) blood and saliva cardiovascular health biomarkers, because the secretion of stress hormones (e.g., cortisol) and biomarkers of inflammation (e.g., C-reactive protein) have been shown to have significant short- and long-term implications on cardiovascular health due to chronic sympathetic nervous system stimulation [11]; and 4) ‘other’ various disease states as indices of cardiovascular health (e.g., heart disease) that have been examined in the context of social discrimination.

### Eligibility criteria

We used the PECOD (Population, Exposure, Comparator outcome, study Design) worksheet to determine *a priori* inclusion criteria (Table 1). Studies were excluded if they met the following *a priori* criteria: a) subjects aged <19 years; b) not published in a peer-reviewed journal; c) not published in English or conducted in the United States; d) included a stigmatized group (e.g., disabled) unrelated to gender, race/ethnicity, age, body weight/obesity, or sexual orientation; or e) did not contain data linking stigmatized groups to a cardiovascular health outcome. This review only included studies conducted in the United States because of the broad range of discrimination types included, and the prevalence of these types of discrimination can vary across different cultures and countries.

**Table 1:** *A priori* criteria for inclusion of studies described by PECOD.

**P****opulation**	Prevalent stigmatized groups in American society, including gender, race/ethnicity, age, body weight/obesity, and sexual orientation aged ≥ 19 years
**E****xposure**	History of discrimination determined via questionnaire / interview or a laboratory stigma exposure
**C****omparator**	Gender (e.g., men vs women); race/ethnicity (e.g., African American vs Caucasian); body weight/obesity (e.g., obese vs normal weight); sexual orientation (e.g., gay/bisexual vs heterosexual)
**O****utcome**	A relationship between discrimination and cardiovascular health indicators including blood pressure, heart rate variability/heart rate, blood and saliva cardiovascular heath biomarkers, and ‘other’ various diseases states as indices of cardiovascular health such as heart disease.
**D****esign**	All study designs were eligible for inclusion except systematic reviews, meta-analyses, and case studies.

### Quality of studies

All non-randomized studies were assessed for methodological quality and risk of bias using the Newcastle-Ottawa Scale (NOS) [22]. The NOS uses a ‘star’ system in which a study is assessed on three subscales including the selection of the study groups, the comparability of the groups, and the ascertainment of either the exposure or outcome of interest. The maximum score a study can receive on each of these subscales is 4, 2, and 3 ‘stars’ respectively. The highest-quality study receives 9 ‘stars’ (S1 Table). All randomized controlled and experimental studies included in the systematic review were assessed for study methodological quality and risk of bias using the 7-item Cochrane Collaboration tool [23]. These criteria assessed several forms of bias including selection, performance, detection, attrition, reporting, and “other.” Studies were given a score of -1, 0, or +1 for each criterion which represented ‘high’, ‘unclear’, or ‘low’ risk, respectively [23]. All scoring on the NOS and the 7-item Cochrane Collaboration tool were conducted by two coders (GAP and ALZ) with 92% and 90% agreement, respectively. All disagreements were discussed and reconciled.

### Data synthesis

The current review was intentionally performed as a systematic review without meta-analysis due to the heterogeneity of the types of measures and samples included in this literature. The systematic review follows a narrative synthesis format which allows for the presentation of important narrative aspects of this literature that have not yet been summarized.

## Results

**Fig 1:**
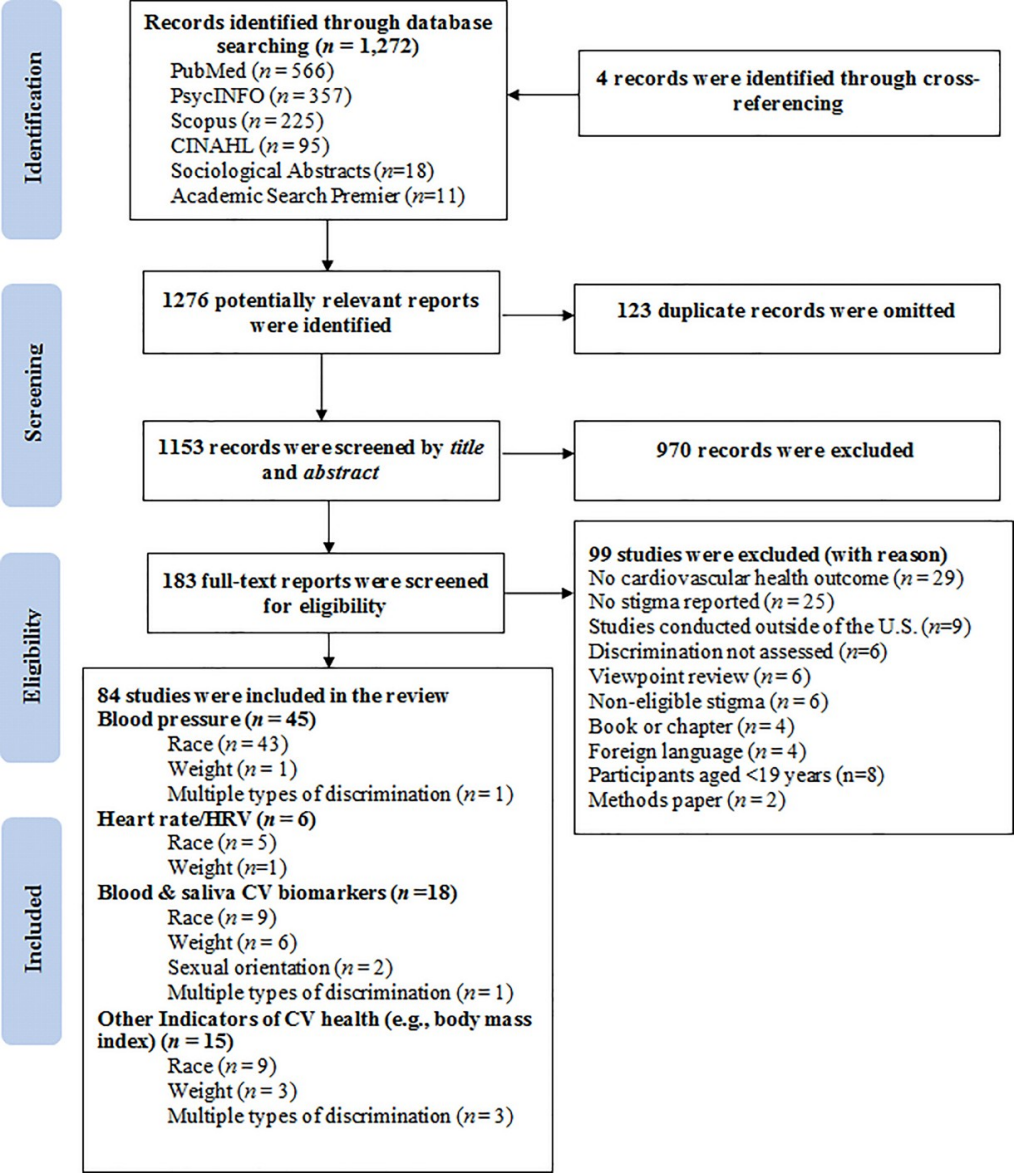
Flow chart detailing the systematic search of potential reports and selection process of included studies (*n*). CV = cardiovascular; HRV = heart rate variability.

**Fig 2:**
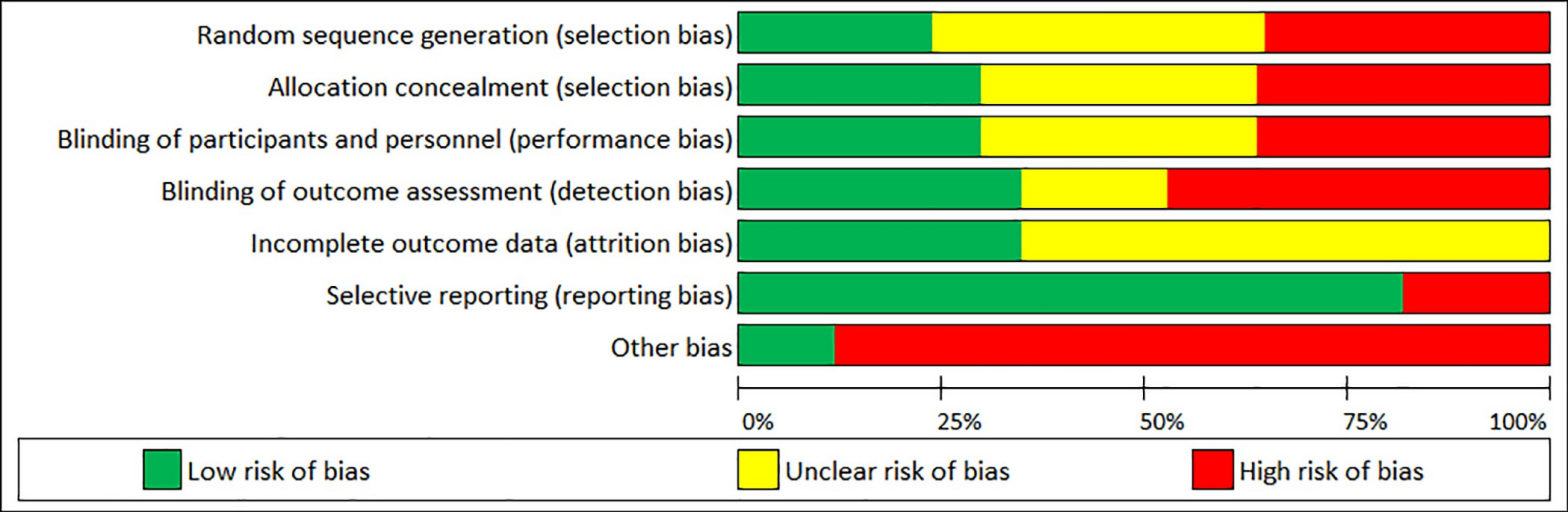
Risk of bias assessment. Results of the risk of bias assessment reported in S1 Table are summarized based on the Cochrane Collaboration tool.

Fig 1 describes the search and selection process which resulted in 1,272 identified records, yielding 84 eligible studies, published between 1984 and 2017. All included studies examined the relationship between social discrimination and one or more cardiovascular health indicators among at least one socially stigmatized group. A summary of study characteristics for the 84 included studies are described in Table 2. A more detailed table of study characteristics (study design, population, measures used to assess discrimination and cardiovascular health indices, and study findings) is presented in S2 Table. Cross-sectional and longitudinal cohort studies included in the systematic review scored an average of 7.5 out of 9 on the NOS (Table 3). Randomized controlled and experimental studies included in the systematic review had an overall average of 35.3% low risk, 37.0% high risk, and 27.7% unclear risk across all 7 domains (Fig 2), with the highest risk shown for “blinding of outcome assessment” (detection bias) and “other bias” (e.g., no power analysis indicated; S1 Table). Summarized below are research findings pertaining to each of the four cardiovascular health indicator categories: 1) BP, 2) HR/HRV, 3) blood/saliva cardiovascular biomarkers, and 4) ‘other’ various disease states as indices of cardiovascular health.

**Table 2:** Characteristics of studies (N = 84) examining the relationship between stigma/discrimination and cardiovascular health outcomes among common socially stigmatized groups.

Author (year)	Study Design	Study Population	Discrimination assessed	Measure of Cardiovascular Health Indices	Length of Study	Significant relationship between stigma/ discrimination & cardiovascular health
**Blood Pressure as Primary Cardiovascular Health Outcome (n = 45)**						
***Race***						
Thayer et al. (2017)	Cross-sectional	American Indian men (n = 21) & women (n = 56)	Daily racial discrimination	Resting BP by mercury sphygmanometer	3 days	Yes
Beatty Moody et al. (2016)	Cross-sectional	Black (n = 318) & Latino (n = 289), men (n = 309) women (n = 298)	Lifetime racism/ethnic discrimination	24-hr ambulatory BP	3 visits within 2 weeks	Yes
Orom et al. (2016)	Cross-sectional	Black (n = 190), Caucasian (n = 1193), Hispanic (n = 120) & other (n = 30) men with prostate cancer	Lifetime racial/ethnic discrimination	Self-reported HTN & resting BP from clinic notes	1 visit	Yes
Dawson et al. (2015)	Cross-sectional	Black (n = 371) & White (n = 231), men (n = 369) & women (n = 233), with type 2 diabetes	Unspecified	SBP & hemoglobin A1c from medical records	1 visit	Yes
Wagner et al. (2015)	Cross-sectional	Black (n = 39) & White (n = 38) women with type 2 diabetes	Lifetime racism/ethnic discrimination	24-hr ambulatory BP	1 visit	Yes
Gregoski et al. (2013)	Cross-sectional	AA (n = 175) & European American (n = 177) men (n = 162), & women (n = 190)	Perceived lifetime discrimination	24-hr ambulatory BP	Data from 1 visit	Yes
Krieger et al. (2013)	Cross-sectional	Black (n = 504) & White (n = 501) men (n = 340) & women (n = 665)	Lifetime & recent discrimination Unconscious associations Structural discrimination	Resting BP by automatic BP monitor	1 visit	No
Chae et al. (2012)	Cross-sectional	AA men (n = 91)	Daily racial discrimination	Resting BP by automatic BP monitor	1 visit	No, main analysis Yes, sub-analysis
Kaholokula et al. (2012)	Cross-sectional	Native Hawaiian men (n = 42) & women (n = 104)	Perceived racism	Resting BP by mercury sphygmomanometer	1 visit	Yes
Mujahid et al. (2011)	Cross-sectional	AA (n = 1,159), Hispanic (n = 415), & Caucasian (n = 1,105) men (n = 1,236) & women (n = 1,443)	Chronic burden Perceived discrimination, Everyday discrimination	Resting BP by automatic sphygmomanometer	1 visit	Yes
Hahm et al. (2010)	Cross-sectional	Asian American men (n = 972) & women (n = 1075)	Perceived discrimination	Self-reported BP	Web based study	No, diabetes only
Krieger et al. (2010)	Cross-sectional	Non-Hispanic black or AA (n = 442) & Caucasian (n = 1018) adults	Exposure to racial discrimination	Self-reported BP	1 visit	No
McClure et al. (2010)	Cross-sectional	Latin American adult men (n = 46) & women (n = 86) immigrants	Perceived discrimination	Resting BP by automatic sphygmomanometer	1 visit	Yes, men only
Smart et al. (2010)	Cross-sectional	Black (n = 31) & White (n = 31) adults	Daily perceived discrimination	24-hr ambulatory BP monitor	1 work day	Yes
Todorova et al. (2010)	Cross-sectional	Puerto Rican men (n = 331) & women (n = 791)	Perceived discrimination	Resting BP by automatic sphygmomanometer	1 visit	Yes
Barksdale et al. (2009)	Cross-sectional	Black American men (n = 64) & women (n = 147)	Perceived racism	Resting BP	1 visit	No
Lewis et al. (2009)	Cross-sectional	AA (n = 2,826) & Caucasian (n = 1,868) adults	Daily perceived discrimination	Resting BP by manual sphygmomanometer	1 visit	Yes
Krieger et al. (2008)	Cross-sectional	Black men (n = 308) & women (n = 158), Latino men (n = 152) & women (n = 115), Caucasian men (n = 182) & women (n = 102), & other ethnicity men (n = 73) & women (n = 45) union workers	Self-reported workplace abuse, racial discrimination, & sexual harassment	Resting BP by automatic sphygmomanometer	1 visit	Yes
Rahman et al. (2008)	Cross-sectional	Predominately black (n = 134) men (n = 63) & women (n = 112)	Self-reported experiences of racial discrimination	Self-reported HTN	1 visit	No
Pointer et al. (2008)	Cross-sectional	Men (n = 63) & women (n = 113) of self-reported African descent	Chronic exposure to racism	Resting BP by automatic sphygmomanometer	1 visit	No
Roberts et al. (2008)	Cross-sectional	AA men (n = 393) & women (n = 717)	Exposure to unfair treatment due to race	Resting BP by automatic monitor	1 visit	Yes, women only & in non-racial discrimination
Salomon & Jagusztyn (2008)	Cross-sectional	White (n = 28), Black (n = 24), & Latino (n = 18) college undergraduate men (n = 21) & women (n = 51)	Perceived discrimination Unfair treatment	Ambulatory BP monitor	1 visit	Yes, Latinos only
Singleton et al. (2008)	Cross-sectional	Black men (n = 11) & women (n = 41)	Exposure & coping responses to racism	24-hr ambulatory BP monitoring	1 visit	Yes
Hill et al (2007)	Cross-sectional	AA men (n = 19) women (n = 21) college students	Perceived racism	24-hr ambulatory BP monitoring	1 visit	Yes
Cozier et al. (2006)	Cross-sectional	Black women (N = 30,330)	Perceptions & experiences of racism	Self-reported BP & subsample by sphygmonometer	Data from 1 visit	No, main analysis Yes, sub-analysis
Brown et al. (2006)	Cross-sectional	AA (n = 934), Caucasian (n = 1549), Chinese (n = 250), Hispanic (n = 286), & Japanese (n = 281) women	Perceived unfair treatment	R&om-zero sphygmomanometer	1 visit	No
Merritt et al. (2006)	RCT	Black men (N = 73)	Stressor experiment	BP & HR by automatic monitor	1 visit	No, main analysis Yes, sub-analysis
Peters (2006)	Cross-sectional	AA men (n = 29) & women (n = 133)	Perceived racism	Resting BP by automatic monitor	1 visit	No
Ryan et al. (2006)	Cross-sectional	Black /AA (n = 190) & Latinos (n = 490)	Perceived racial/ethnic discrimination	BP measured using a digital BP monitor after survey	1 visit	Yes
Davis et al. (2005)	Cross-sectional	AA men (n = 160) & women (n = 196) with (n = 174) & without HTN (n = 182)	Perceived racial discrimination	BP by mercury sphygmomanometer	1 visit	No
Din-Dzietham et al. (2004)	Cross-sectional	AA (n = 356) men (n = 160) & women (n = 196)	Perceived responses to general stress & racism	Resting BP by mercury sphygmomanometer	1 visit	Yes
Peters (2004)	Cross-sectional	AA men (n = 29) & women (n = 133)	Perceived racism	Resting BP by automatic monitor	1 visit	No
Clark & Adams (2004)	Experimental	Black women (N = 117) college students	Ethnicity stressor experiment Perceptions of interethnic group racism Active coping	BP by automatic monitor	1 visit	Yes
Clark (2003)	Experimental	Black men (N = 64) college students	Math stressor experiment Perceived racism Social support assessed	BP by automatic monitor	1 visit	Yes
Steffen et al. (2003)	Cross-sectional	AA men (n = 30) & women (n = 39)	Perceived racism	Resting BP by mercury sphygmomanometer Daytime ambulatory BP	3 visits, each 1 week apart for clinic BP	Yes
Blascovich et al. (2001)	RCT	AA (n = 20) & European-American (n = 19) university students	Stressor experiment on stereotypes	MAP by automatic BP monitor	1 visit	Yes
Fang & Myers (2001)	Experimental	AA (n = 31) & Caucasian (n = 31) undergraduate men	Racial video experiment Emotions	Automatic BP monitor	1 visit	Yes, but no differences by race
Guyll et al. (2001)	Experimental	AA (n = 101) & European American (n = 262) women	Social stressor speech experiment Experiences of mistreatment & discrimination	Automatic BP monitor	1 visit	Yes
Clark (2000)	Cross-sectional	AA graduate & undergraduate women (N = 39)	Speech stressor experiment Perceptions of racism, psychological, & coping responses to racism	Automatic BP monitor	1 visit	Yes
Krieger & Sidney (1996)	Cross-sectional	Black (n = 1,974) & White (n = 2,112) men (n = 1,837) & women (n = 2,249)	Racial discrimination & unfair treatment	Resting BP by sphygmomanometer	1 visit	Yes
McNeilly et al. (1995)	RCT	AA women (N = 30) aged 18–33 years, with normal BP	Racist & non-racist debate stressor experiment	Resting BP by automatic BP monitor	1 visit	Yes
Armstead et al. (1989)	RCT	Black men (n = 12) & women (n = 15) college students	Racist film experiment	BP with a sphygmomanometer	1 visit	Yes
James et al (1984)	Cross-sectional	Black men (N = 112)	Perceived racism hindrance to job success	BP by auscultation	1 visit	No
***Weight***						
Major et al. (2012)	Experimental	Women (N = 99) who perceived themselves as overweight	Video/audio tape speech experiment on dating	BP by automatic BP monitor; MAP reactivity calculated	1 visit	Yes
***Multiple Types of Stigma/Discrimination***						
Krieger, N. (1990)	Cross-sectional	AA (n = 51) & Caucasian (n = 50) women	Response to unfair treatment & gender & race discrimination	Self-reported BP	1 phone interview	Yes
**Heart Rate / Heart Rate Variability as Primary Cardiovascular Health Outcome (n = 6)**						
***Race***						
Hill et al. (2017)	Cross-sectional	AA men (n = 43) & women (n = 56)	Perceived racial discrimination	HRV via ECG	1 visit	Yes
Kemp et al. (2016)	Cross-sectional	Brown (n = 3,502), White (n = 6,467), & Black (n = 2020) men (n = 5,468) & women (n = 6,521)	Perceived discrimination	HRV via ECG	1 visit	Yes
Hoggard et al. (2015)	Experimental	AA women (N = 42) college students	Racial discrimination dialogue experiment	HRV via ECG	2 days	Yes
Wagner et al. (2013)	Cross-sectional	Black (n = 16) & White (n = 16) women with type 2 diabetes	Public speaking stressor experiment Racial discrimination Racial attribution	HRV via ECG; Cortisol & norepinephrine via serum; BP & HR via BP monitor	1 visit	Yes
Utsey et al. (2007)	Cross-sectional	AA undergraduate college student men (n = 83) & women (n = 132)	Lifetime experience of race-related stress	HRV & HR measured via Heart Rate Monitor	1 visit	Yes, men only
***Weight***						
Kube et al. (2016)	Experimental	Women with (n = 14) & without obesity (n = 14)	Simplified version of MID task Adaptation of SID task Face rating Negative social experiences	HRV measured via ECG; HR estimated in 500-ms intervals	1 visit	Yes
**Blood & Saliva Cardiovascular Biomarkers (n = 18)**						
***Race***						
Lucas et al. (2017)	Experimental	AA men (n = 21) & women (n = 64)	Psychosocial stress experiment Perceived racial discrimination Racial identity	Alpha-amylase, cortisol, DHEA, & C-reactive protein	1 visit	Yes
Lucas et al. (2016)	Experimental	AA men (n = 36) & women (n = 82) aged 31.6±13.8 years	Psychosocial stress experiment Attributions of racism Justice beliefs	Salivary cortisol & C-reactive protein	1 visit	Yes
Giurgescu et al. (2016)	Cross-sectional	AA women (N = 96) during second trimester of pregnancy	Perceived lifetime discrimination	Plasma interleukin-1β, 2, 4, 6, 8, & 10	1 visit	Yes
Brody et al. (2015)	Longitudinal	AA (N = 160)	Perceived racial discrimination	Serum interlueken-1β, 6, 8, & 10, & tumor necrosis factor-α & interferon	3 years	Yes
Zeiders et al. (2014)	Cross-sectional	Caucasian/White (n = 76), AA/Black (n = 11), Asian (n = 8), Hispanic/Latino (n = 19), Pacific Isl&er (n = 1), multiethnic /multiracial (n = 15), & other (n = 10) men (n = 38) & women (n = 102)	Perceived discrimination assessed	Salivary cortisol	3 days	Yes
Cunningham et al. (2012)	Cross-sectional	Black (n = 1,515) & White (n = 1,821) men (n = 1,477) & women (n = 1,859)	Perceived experiences of racial/ethnic discrimination	C-reactive protein from blood	20 years	Yes, women only
Lewis et al. (2010)	Cross-sectional	AA men (n = 86) & women (n = 210)	Daily discrimination	Plasma C-reactive protein	1 visit	Yes
Cooper et al. (2009)	Cross-sectional	Black (n = 51) & White (n = 65) men (n = 57) & women (n = 59)	Exposure to discrimination	Plasma endothelin-1	1 visit	Yes
Tull & Chambers (2001)	Cross-sectional	Black men (n = 13) & women (n = 14) with type 2 diabetes aged 58.7±11.2 years, & Black men (n = 24) & women (n = 31) controls without type 2 diabetes	Measurement of internalized racism not specified	Fasting blood glucose	1 visit	Yes
***Weight***						
Rodriguez et al. (2016)	RCT	Men (n = 26) & women (n = 83) university students	“Fat suit” experiment Anger, anxiety, & depression Hurt feelings Self-esteem Antifat attitudes	Salivary cortisol	1 visit	No, main analysis Yes, sub-analysis
Himmelstein et al. (2015)	RCT	Undergraduate women (N = 110) aged 19.8±4.8 years	Weight stigma clothes shopping experiment Self-perceived body weight Negative affect	Salivary cortisol	1 visit	Yes
Schvey et al. (2014)	RCT	Lean (n = 69) & overweight (n = 54) adult women	Weight-based discrimination video exposure experiment Positive & negative effect Depressive symptoms Fat phobia Perceived stress Emotional reactions	Salivary cortisol	1 visit	Yes
Sutin et al. (2014)	Cross-sectional	Overweight or obese (BMI >25) men (n = 3,179) & women (n = 4,215)	Perceived discrimination assessment not specified	High sensitivity C-reactive protein via finger prick	1 visit	Yes
Tomiyama et al. (2014)	Cross-sectional	Subsample of overweight or obese women (N = 47)	Exposure of weight stigma Consciousness of weight stigma	Salivary cortisol Oxidative stress via blood Adiposity via DEXA	4 days	Yes
Tsenkova et al. (2011)	Cross-sectional	Men (n = 403) & women (n = 535)	Perceived daily weight discrimination	Nondiabetic glycemic control by HbA1c	Data from 1 time point	Yes
***Sexual Orientation***						
Doyle & Molix (2016)	Cross-sectional	Gay men (n = 78) & Lesbian women (n = 21)	Perceived discrimination	Salivary interleukin-6	1 visit	Yes, gay men only
Hatzenbuehler & McLaughlin (2014)	Cross-sectional	Lesbian/gay (n = 42) & bisexual (n = 32) men (n = 34) & women (n = 40)	Experiment: Participants exposed to a laboratory stressor & social-evaluative threat task Perceived discrimination	Salivary cortisol	1 visit	Yes
***Multiple Types of Stigma/Discrimination***						
Reynolds et al. (2015)	Cross-sectional	AA (n = 399) & Other (n = 203) men (n = 369) & women (n = 233) with type 2 diabetes	Perceived race/ethnic, level of education, sex/gender, & language discrimination	Glycemic control via HbA1c	Data from 1 time point	No, race, gender, language Yes, education Ed
**Other Indicators of Cardiovascular Health (n = 15)**						
***Race***						
Everson-Rose et al. (2015)	Longitudinal	White (39%), Black (26.4%), Chinese (12.2%), & Hispanic (22.3%) men (n = 3,072) & women (n = 3,436)	Perceived lifetime discrimination assessed Perceived everyday discrimination	Incident myocardial infarction, resuscitated cardiac arrest, coronary revascularization, definite angina, fatal or nonfatal stroke, & CVD death	10.1 years	Yes
Neblett et al. (2013)	Cross-sectional	AA men (n = 45) & women (n = 60) college students	Race-related beliefs & attitudes Experimental session with racism analogues	Respiratory sinus arrhythmia via spectral analysis; Cardiac pre-ejection period via onset of ECG; HRV via ECG	1 visit	Yes
Wagner et al. (2013)	Cross-sectional	White (n = 94) & minority (n = 19) women with (n = 49) & without (n = 64) diabetes	Mental arithmetic & harassment experiment Perceived lifetime discrimination Perceived stress	Flow-mediated endothelial function; Peak HR & peak BP via semi-automatic digital manometer; Vasoconstriction via ultrasound	1 visit	Yes
Chae et al. (2012)	Cross-sectional	Black American men (n = 1,847) & women (n = 3,175)	Racial discrimination Mood disorder	History of CVD via self-report	Data from 1 time point	Yes
Mwendwa et al. (2011)	Cross-sectional	AA women (N = 110)	Perceived racism a Perceived stress	Weight & height via balance scale	1 visit	Yes
Peek et al. (2011)	Cross-sectional	Non-Hispanic White (n = 1,591), AA (n = 416), Hispanic (n = 87), Multiracial (n = 49), & Other (n = 95) men (n = 1,132) & women (n = 1,106)	Self-reported discrimination in healthcare	Diabetes quality of care, diabetes self-management, & diabetes complications	Data from 1 time point	Yes
Cardarelli et al. (2010)	Cross-sectional	Non-Hispanic White (n = 142), AA (n = 167), Hispanic (n = 193)	Perceived racial discrimination & response to unfair treatment	CAC via a16-slice MSCT scan	1 visit	Yes
Thomas et al. (2006)	Cross-sectional	White (n = 76) & Black (n = 46) men (n = 65) & women (n = 57)	Experiences of ethnicity	Pressor Responses to Phenylephrine via ECG	1 visit	Yes
Troxel et al. (2003)	Cross-sectional	AA (n = 109) & Caucasian (n = 225) women	Racial discrimination	Carotid ultrasound	1 visit	Yes
***Weight***						
Puhl et al. (2017)	Longitudinal	Underweight, normal weight, overweight, & obese, men (n = 788) & women (n = 1,042)	Weight-based teasing by peers	Changes in BMI via self-reported height & weight & self-report unhealthy weight control	Data from 2 time points over 15 years	Yes, with differences across gender & teasing source
Jackson et al. (2014)	Longitudinal	Normal, overweight, & obese, men (n = 1,216) & women (n = 1,728)	Perceived weight discrimination	Changes in weight & waist circumference objectively measured	Data from 2 time points over 5 years	Yes
Sutin & Terracciano (2013)	Longitudinal	Obese & non-obese men (n = 2,549) & women (n = 3,608)	Perceived everyday weight discrimination	Changes in weight & waist circumference objectively measured	4 years	Yes
***Multiple Types of Stigma/Discrimination***						
Udo & Grilo (2017)	Longitudinal	Adult men (n = 12,011), & women (n = 14,981)	Perceived experiences with discrimination due to weight, race/ethnicity, & gender	CVD assessed via self-reported atherosclerosis, HTN, myocardial infarction, & all other heart diseases	Data from 2 time points over 3 years	Yes
Clark & Hill (2009)	RCT	Normal, overweight, & obese AA men (n = 15) & women (n = 33) college students	Racism video tape experiment	Cardiac output, stroke volume, HR, & BP	1 visit	No
Lewis et al. (2006)	Cross-sectional	AA women (N = 181)	Perceived race, ethnicity, age, income level, language, physical appearance, sexual orientation, & other types of discrimination	CAC via electron beam tomographic scans; Framingham Risk Score calculated via st&ard techniques	Data averaged over 5 years	Yes

Abbreviations: AA = African American; AHA = American Heart Association; BMI = body mass index; BP = blood pressure; CAC = coronary artery calcification; CARDIA = Coronary Artery Risk Development in Young Adults study; CHD = coronary heart disease; CVD = cardiovascular disease; DBP = diastolic blood pressure; DEXA = dual-energy x-ray absorptiometry; DHEA = Dehydroepiandrosterone-sulfate; DODARS = Dominica Obesity and Diabetes Risk Survey; EAT-IV (Eating and Activity in Teens and Young Adults); ECG = electrocardiogram; ELSA = The Brazilian Longitudinal Study of Adult Health; FNS = Fourth National Survey of Ethnic Minorities; HOMA = homeostasis model assessment; HR = heart rate; HRV = heart rate variability; HTN = hypertension; JNC = Joint National Committee; MAP = mean arterial pressure; MESA = Multi-Ethnic Study of Atherosclerosis) MID = monetary incentive delay; Multi-MESA = Ethnic Study of Atherosclerosis; NESARC = National Epidemiologic Survey of Alcohol and Related Conditions; NSAL = National Survey of American Life; NZHS = New Zealand Health; RCT = randomized controlled trial; SBP = systolic blood pressure; SID = social incentive delay; Survey; SWAN = Study of Women’s Health Across the Nation; TSST = Trier Social Stress Test; WC = waist circumference

**Table 3:** Summary of study quality scores of the included cross-sectional and longitudinal cohort studies assessed by the Newcastle-Ottawa Scale (NOS).

Cardiovascular health risk factor Type of discrimination (N = 67 Studies)	Mean quality score for selection (max 4)	Mean quality score for comparability (max 2)	Mean quality score for selection / exposure (max 3)	Total mean quality score (max 9)
Blood Pressure (*n* = 36)				
Race (*n* = 35)	2.8	2.0	1.7	6.5
Multiple types (*n* = 1)	4.0	2.0	3.0	9.0
Heart rate variability / heart rate (*n* = 4)				
Race (*n* = 4)	3.3	1.7	2.2	7.2
Blood/saliva cardiovascular biomarkers (*n* = 13)				
Race (*n* = 7)	3.1	1.7	2.3	7.1
Weight (*n* = 3)	2.7	2.0	2.3	7.0
Sexual orientation (*n* = 2)	2.5	2.0	3.0	7.5
Multiple types (*n* = 1)	3.0	2.0	3.0	8.0
Other cardiovascular health risk factors (*n* = 14)				
Race (*n* = 9)	3.6	2.0	2.7	8.2
Weight (*n* = 3)	3.0	2.0	3.0	8.0
Multiple types (*n* = 2)	2.5	2.0	2.0	6.5
**Totals**	**3.1**	**1.9**	**2.5**	**7.5**

Note. The scoring for each individual study can be found in S1 Table.

### I: Social discrimination and BP

Forty-five studies examined BP and different types of social discrimination including race (*n* = 43), weight (*n* = 1), and multiple types of discrimination (*n* = 1).

#### Racial discrimination

Subsamples (*n* = 72) within the 43 studies examining race consisted primarily of African American (AA)/Black (56%), followed by Caucasian (22%), Hispanic/Latino (16%), Asian American (4%), and American Indian (1%). Cross-sectional studies (*n* = 35) assessed race discrimination using a variety of self-report questionnaires and BP was measured with a range of methods (S2 Table). Of these cross-sectional studies, 22 [24–37,37–42] found significant associations between racial discrimination and BP in their primary analysis. Two cross-sectional studies [43,44] did not find a significant association between racial discrimination and BP in their primary analysis among the entire sample. However, they did find a positive association for their secondary outcomes which included an association between racial discrimination among only participants who immigrated to the US [44] and those who reported high rates of implicit racial discrimination [43]. Eleven cross-sectional studies [45–56] found no association between racial discrimination and BP.

Four studies [57–60] examined the relationship between racial discrimination among AAs and BP using experimental designs, with two studies [58, 60] using a Caucasian comparison group. All four studies used different tasks including speaking [57], mathematical [59], video [58], and a social stressor [60] to elicit BP responses measured via automated BP monitor. All four studies found the BP response tasks elicited an increased BP response among AAs during and following the stressors, with one study not finding differences by race [58].

Four studies [61–64] examined the relationship between racial discrimination and BP using randomized controlled trial (RCT) designs, all focused on AA/blacks. In these studies, racial stigma was induced in experimental conditions via exposure to video tapes (*n* = 2), audio tape (*n* = 1), and verbal debate (*n* = 1). The most common assessment of BP was an automatic BP monitor (*n* = 3). To put these experimental findings into clinical context, it is important to consider that an increment increase in systolic BP (SBP) of 20 mmHg or diastolic BP (DBP) of 10 mmHg above 115/75 mmHg doubles the risk of CVD [65–67]. However, individuals with elevated BP (i.e., SBP ≥120 and DBP <80 mmHg) [68] or hypertension (i.e., SBP ≥130 or DBP ≥80 mmHg) [68] have a higher risk of CVD [69], therefore, smaller increases may be clinically meaningful. For example, one RCT [64] found that racial discrimination significantly increased SBP by 1.4 mmHg and DBP by 2.6 mmHg, while another RCT [63] found significant increases vs control in SBP ranging from 5.3 to 30.3 mmHg and DBP ranging from 7 to 18mmHg in the group receiving racist provocation [63]. Importantly, a RCT [61] of black normotensive men found elevated BP in response to racially ambiguous stimuli, suggesting that even subtle forms of racism (not just exposure to blatant discrimination) can induce these responses.

#### Weight discrimination

Only Major and colleagues [12] examined the effect of a social stressor to activate concerns about weight stigma on mean arterial pressure (MAP) using a randomized experimental design. Ninety-nine women aged 18.8±1.3 years who perceived themselves as overweight with a BMI of 27.4±5.6 kg∙m^2^ were randomized to a weight salient group who believed they were being viewed by others while giving a video-taped speech, or a neutral group who were informed that their body size would not be visible while giving an audio-taped speech. Continuous readings of BP were measured during both speech types. Higher BMI was associated with increased MAP among individuals who believed they were being video-taped (visible to others) compared to those giving an audio-taped speech. That is, for every 1 kg∙m^2^ increase in BMI, MAP increased by .25 mmHg among individuals who believed they were giving the speech.

#### Multiple types of discrimination

Krieger [70] examined the relationship between BP and both gender and race discrimination among AA (*n* = 51) and Caucasian (*n* = 50) women aged 20–80 years. Higher internalized unfair treatment and the recounting of less racist or sexist incidents associated with higher BP, while there was no association found for Caucasian women.

### II: Social discrimination and heart rate variability/heart rate

Six studies examined the relationship between HRV/HR and social discrimination including racial (*n* = 5) and weight (*n* = 1).

#### Racial discrimination

Subsamples (*n* = 8) within the five studies examining race consisted primarily of AA/Black (63%), followed by Caucasian/White (25%), and Hispanic/Latino (13%). Four studies [15,71–73] examined the relationship between racial discrimination and HR/HRV using cross-sectional designs, and each assessed racial discrimination using a different self-report questionnaire (S2 Table). All four cross-sectional studies found a negative association [15,71–73] between racial discrimination and HRV, such that increased racial discrimination was associated with decreased HRV. Three [71–73] of these cross-sectional studies found this relationship for high frequency HRV, while one did not report the HRV frequency measured in their results [15]. High frequency HRV is associated with respiration, representing respiratory sinus arrhythmia [74], and is a reflection of parasympathetic or vagal activity. Thus, these studies indicate that increased racial discrimination is associated with high frequency HRV, which has previously been linked to panic, stress, and anxiety [75].

Hoggard and colleagues [76] examined the relationship between racial discrimination and HRV using a randomized experimental design among 42 AA women. The women were randomized to participate in a scripted racial discrimination dialogue session led by either an AA or a European American ‘perpetrator.’ They were then asked to reflect on the session the following day and manipulation checks indicated that participants in both groups experienced the event as being equally discriminatory. The women who were insulted by the European American ‘perpetrator’ during the dialogue exhibited lower (.84 milliseconds) mean squared differences in successive R-R intervals representing a decrease in HRV (measured via electrocardiogram) and greater sympathetic nervous system activity, and also had higher HR during the reflection visit. However, the women who were insulted by the AA ‘perpetrator’ exhibited an increase in HRV. These results indicate that intergroup racial discrimination may have both momentary and prolonged effects on cardiac activity, while within group racial discrimination did not show negative effects.

#### Weight discrimination

Kube and colleagues [77] examined the relationship between weight discrimination and HRV measured via electrocardiogram in a cross-sectional study. Women with (*n* = 14) and without (*n* = 14) obesity aged 25.3±2.9 years participated in a monetary and social incentive delay task in which they anticipated and received positive, negative, and neutral outcomes in the form of money or facial expressions. Women with obesity demonstrated diminished HR responses to negative social outcomes compared to controls. The authors suggested that the diminished HR responses found during negative social feedback may be due to reduced salience (i.e., lack of importance or prominence) since HR responses may depend on the incentive salience of the stimuli [78]. Differences in cardiac responses in women with obesity were moderated by weight-related teasing experiences.

### III: Social discrimination and blood/saliva cardiovascular stress biomarkers

Eighteen studies examined the relationship between blood/saliva cardiovascular biomarkers and social discrimination including race (*n* = 9), weight (*n* = 6), sexual orientation (*n* = 2), and multiple types of discrimination (*n* = 1).

#### Racial discrimination

Subsamples (*n* = 17) within the nine studies examining race were primarily AA/Black (53%), followed by Caucasian/White (19%), Hispanic/Latino (13%), Pacific Islander (5%), Multiethnic/Multiracial (5%), and other (5%). Six cross-sectional studies [14,79–83] examined the relationship between racial discrimination and blood/saliva cardiovascular biomarkers, using a variety of measures (S2 Table). All six studies found that higher racial discrimination was associated with higher levels of blood/saliva biomarkers including cortisol, [14] C-reactive protein [80,81], interleukin 4 and 6 [79], endothelin-1 [82], and blood glucose [83].

Lucas and colleagues [16] examined the relationship between racial discrimination and saliva cardiovascular biomarkers using a randomized experimental design. Black men (*n* = 36) and women (*n* = 82) aged 31.6 years completed baseline measurements of justice beliefs (e.g., rules, process) followed by a social-evaluative stressor task. During the task, participants were randomly given either high or low levels of distributive and procedural (decision process) justice. Oral fluids were assayed for cortisol (stress hormone) and C-reactive protein (marker of inflammation) at baseline and the recovery phases of the stressor. The cortisol and C-reactive protein responses to low distributive justice were significantly higher when procedural fairness was low vs high among blacks with a strong belief in justice and perceived racism (53.4 vs 26.4 mg/dL and 665.9 vs 526.3 thousands of pg/mL, respectively). Excess secretion of cortisol is associated with cardiovascular health issues including elevated BP, truncal obesity, dyslipidemia, and insulin resistance [84], while c-reactive protein is a risk marker for CVD due to its role in inflammation and atherosclerosis [85]. Excess levels of C-reactive protein and cortisol during the recovery phase may have been due to rumination leading to the participant’s inability to disengage from the stressor, thus prolonging the recovery periods [86].

Another experimental study [87] induced mild psychosocial stress using the Trier Social Stress Test, and found that when racial identity was strong, perceived discrimination was associated with low hypothalamic-pituitary-adrenal axis activity at baseline (*β*’s = .68-.72, *p*<0.001), low stress mobilization during the test (*β*’s = .68-.72, *p*<0.001), and an increase in salivary C-reactive protein (*β*’s = .72-.94, *p* ≤ .002). Hypothalamic-pituitary-adrenal axis dysfunction is a predictor of CVD [88], while the increase in C-reactive protein during recovery indicates an inflammatory response to the test. A 3-year longitudinal study [89] found that young AA men and women exposed to high levels of racial discrimination predicted elevated cytokine levels (*p*<0.001).

#### Weight discrimination

Three cross-sectional studies [90–92] found that increased weight discrimination was associated with higher levels of blood/saliva cardiovascular stress biomarkers including C-reactive protein [92], cortisol [90], and HbA_1c_ [91]. HbA_1c_ is the average of blood glucose levels over approximately 8–12 weeks, and high levels of HbA_1c_ has been associated with poor cardiovascular health [93]. Three RCTs [94]; [95,96] examined the relationship between weight discrimination and blood/saliva cardiovascular biomarkers. Himmelstein and colleagues [95] found that participants who were exposed to experimentally manipulated weight stigma exhibited sustained cortisol elevation post-manipulation compared to individuals who were not exposed. Schvey and colleagues [96] found that participants who watched a 10-minute video containing weight-based stigmatizing scenarios exhibited more sustained cortisol reactivity (~ -.73 to -.78 mg/dL) compared to participants watching a neutral video (~ -.71 to -.84 mg/dL), independent of weight status. Finally, Rodriguez and colleagues [94] found that wearing a ‘fat suit’ did not influence participants’ levels of cortisol reactivity between the experiment and control groups.

#### Sexual orientation discrimination

Two cross-sectional studies examined the relationship between sexual orientation discrimination and blood/saliva cardiovascular biomarkers [salivary interleukin-6 [97] and cortisol [98]], both among samples of gay men and lesbian women. Both [97,98] studies found that sexual orientation discrimination (measured using self-report surveys or a social-evaluative threat task) was positively associated with levels of these blood/saliva biomarkers. However, one study [97] found that perceived discrimination was only predictive of higher levels of interleukin-6 for gay men (not women) who downplayed their sexual identity. Interleukin-6 is a pro-inflammatory cytokine and serves an essential role in the pathophysiology of CVD [99].

#### Multiple types of discrimination

One cross-sectional study [100] examined the relationship between multiple types of discrimination and blood/saliva cardiovascular biomarkers. Self-reported race, level of education, sex/gender, and language discrimination were assessed among AA men and women with type 2 diabetes, and found that only education discrimination was associated with glycemic control.

### IV: Social discrimination and other cardiovascular health indicators

Fifteen studies examined the relationship among other cardiovascular health indicators (e.g., history of CVD) and social discrimination, including discrimination based on race (*n* = 9), weight (*n* = 3) and multiple types of discrimination (*n* = 3).

#### Racial discrimination

Subsamples (*n* = 11) within the nine studies examining race were primarily AA/Black (36%) and Caucasian (36%), followed by Hispanic/Latino (18%), and Multiethnic/Multiracial (9%). Of these, eight studies [101–108] used cross-sectional designs and examined the relationship among racial discrimination and other cardiovascular health indices including history of CVD (*n* = 1), BMI (*n* = 1), coronary artery calcification (CAC; *n* = 1), the pressor response (*n* = 1), carotid ultrasound (*n* = 1), diabetes (*n* = 1), respiratory sinus arrhythmia (*n* = 1), and endothelial function (*n* = 1), with a variety of self-report questionnaires to assess race discrimination (S2 Table). One study [101] administered an experimental session with racism analogues, while another administered a mental arithmetic experiment with harassment [103]. All eight cross-sectional studies found associations [101–108] among racial discrimination and cardiovascular health indices. Everson-Rose and colleagues [109] examined the relationship between racial discrimination and cardiovascular events in a 10-year longitudinal study among White, Black, Chinese, and Hispanic men and women. Men and women who self-reported lifetime racial discrimination had a 38% greater risk of incident CVD than those reporting no lifetime racial discrimination. Everyday racial discrimination was associated with incident CVD in men only.

#### Weight discrimination

Two longitudinal studies [110,111] demonstrated a positive association between perceived weight discrimination and increases in weight and waist circumference. Sutin and Terracciano [111] found that participants who experienced weight discrimination were ~2.5 times more likely to become obese over time and ~3 times more likely to remain obese at follow-up compared to those who had not experienced discrimination. Jackson and colleagues[110] observed this association with increased odds of becoming obese over time, but the odds of remaining obese did not differ by experiences of weight discrimination. A third longitudinal study [112] found that weight-based teasing in adolescence predicted obesity in adulthood.

#### Multiple types of discrimination

Clark and Hill [113] examined the effects of body mass measured by BMI on cardiovascular reactivity (e.g., cardiac output, HR, and BP) to racism among normal, overweight, and obese AA men (*n* = 15) and women (*n* = 33), aged 19 years using a RCT design. Participants viewed a video scene depicting racism and a neutral scene in randomized order. Participants with obesity had greater stroke volume and cardiac output following the video exposure than normal weight participants, demonstrating greater cardiac reactivity among individuals with obesity, an indicator of poor cardiovascular health. Furthermore, the women with obesity had the largest and the men with obesity had the smallest drop in HR from the stressor period to recovery, representing sustained cardiovascular reactivity among the men.

Lewis and colleagues [114] examined the relationship among multiple types of discrimination (e.g., race/ethnicity, sexual orientation) and CAC (i.e., calcium in the arteries) among 181 AA women aged 50.2±2.8 years using a cross-sectional design. Chronic exposure to all discrimination types was associated with CAC, and CVD risk factors. Recent discrimination was marginally associated with the presence of CAC, while persistent exposure to racial/ethnic discrimination was largely associated with CAC. Similarly, in a 3-year longitudinal study, Udo and Grilo [115] found that perceived weight and racial discrimination were associated with a greater likelihood of reporting myocardial infarction, atherosclerosis, and minor heart conditions among adult men (*n* = 12,011) and women (*n* = 14,981) aged 49.2±16.4 years.

## Discussion

This review aimed to provide an overview of the scientific evidence linking discrimination and cardiovascular health indicators among socially stigmatized groups. Overall, there was support for the CDC [5] and the WHO’s [6] recognition of stigma as a public health priority because of its potential to accelerate disease processes, with 86% of studies in the current review concluding that there is a significant relationship between discrimination reported by stigmatized groups and indicators of adverse cardiovascular health. However, there are varying strengths of evidence supporting this relationship based on study design and types of discrimination and cardiovascular health indicator (Table 4). The majority of included studies were cross-sectional (61 of 84); thus, a causal relationship between social discrimination and cardiovascular health outcome cannot be determined in many cases. Longitudinal, RCT designs should be implemented to better establish sequences of events which can lead to different relationships between social discrimination and cardiovascular health over time. Examining this relationship both acutely and chronically will provide a better understanding of the role that social discrimination plays in the disease pathology and progression of CVD.

**Table 4:** Summary of evidence examining the links between discrimination and cardiovascular health among socially stigmatized groups.

Cardiovascular health risk factor Type of discrimination (N = 84 Studies)	Cross-sectional (correlational) Total (+ studies)	Experimental Total (+ studies)	Longitudinal Total (+ studies)	RCT Total (+ studies)
Blood Pressure (*n* = 45)				
Race (*n* = 43)	35 (24)	4 (4)	0	4 (4)
Weight (*n* = 1)	0	1 (1)	0	0
Gender (*n* = 0)	0	0	0	0
Sexual orientation (*n* = 0)	0	0	0	0
Age (*n* = 0)	0	0	0	0
Multiple types (*n* = 1)	1 (1)	0	0	0
Heart rate variability / heart rate (*n* = 6)				
Race (*n* = 5)	4 (4)	1 (1)	0	0
Weight (*n* = 1)	0	1 (1)	0	0
Gender (*n* = 0)	0	0	0	0
Sexual orientation (*n* = 0)	0	0	0	0
Age (*n* = 0)	0	0	0	0
Blood/saliva cardiovascular biomarkers (*n* = 18)				
Race (*n* = 9)	6 (6)	2 (2)	1 (1)	0
Weight (*n* = 6)	3 (3)	0	0	3 (3)
Gender (*n* = 0)	0	0	0	0
Sexual orientation (*n* = 2)	2 (2)	0	0	0
Age (*n* = 0)	0	0	0	0
Multiple types (*n* = 1)	1 (1)	0	0	0
Other cardiovascular health risk factors (*n* = 15)				
Race (*n* = 9)	8 (8)	0	1 (1)	0
Weight (*n* = 3)	0	0	3 (3)	0
Gender (*n* = 0)	0	0	0	0
Sexual orientation (*n* = 0)	0	0	0	0
Age (*n* = 0)	0	0	0	0
Multiple types (*n* = 3)	1 (1)	0	1 (1)	1 (0)
**Totals**	61 (50)	9 (9)	6 (6)	8 (7)

**+** studies denotes the number of studies that demonstrated significant findings linking stigma/discrimination and cardiovascular health; RCT = Randomized controlled trial

The NOS has not yet established a criteria to determine what is considered a ‘high,’ ‘moderate’, or ‘low’ quality study [22]. However, the overall mean score on the NOS for the included studies was 7.5 out of 9 ‘stars’, and previously published systematic reviewers using the NOS have determined a score ≥ 7 ‘stars’ as a score that constitutes a high-quality study [116]. The Cochrane Collaboration risk of bias assessment tool [23] indicated the need to improve the blinding of outcome assessments as well as other forms of bias (such as performing power analysis) to determine appropriate sample size among randomized controlled and experimental studies. This tool also indicated the need for better transparency in reporting random sequence generation for sample allocation as well as reporting data attrition rates (S1 Table).

In addition, a majority of the studies (66 of 84) examined the relationship between cardiovascular health and racial discrimination among primarily AA/blacks, indicating a lack of studies examining other racial/ethnic minorities and different types of discrimination. In particular, age discrimination was only examined in two studies [114,117] and gender discrimination in two studies[100,117]. Studies examining sexual orientation only included samples of gay or lesbian participants but not bisexual, transgender, or questioning adults. It is important to further examine vulnerability to CVD among more diverse samples within stigmatized groups as well as other common types of discrimination such as gender, age, and weight.

A wide variety of measures were used across the 84 included studies to assess social discrimination and cardiovascular health. In total, 23 self-report questionnaires were used to assess social discrimination, five different methods were used to assess BP, two to assess HRV, and three to assess HR (S2 Table). Among the 15 studies that included an experimental manipulation/stressor, 11 had stressors that included a stigmatizing stressor [57,58,61–64,95,96,113,118,119], while four used a physical stressor intended to increase reactivity (e.g., subtraction test), but did not include a stigmatizing stressor [12,59,60,98]. Therefore, it is difficult to speculate whether these studies may have had a different finding if they used stressors that included stigma. The use of diverse measures makes it difficult to compare and confirm validity and reliability of results in this literature. Therefore, future work is needed to build consensus around best practices for measurement approaches to assess both discrimination and cardiovascular health indices.

There were also a wide range of sample sizes (S2 Table) used among studies examining the relationship between social discrimination and BP (*N* range = 27–6,112), HRV/HR (*N* range = 28–23,978), blood/saliva biomarkers (*N* range = 47–7,394), and other indices of cardiovascular health (*N* range = 48–26,992). These large ranges indicate the possibility of underpowered studies and sample size bias among studies.

Twelve studies did not find an association between social discrimination and cardiovascular health, and 11 [45–54,56] of these 12 studies examined racial discrimination and BP. These studies had several methodological weaknesses that may help explain their negative findings. First, all 11 studies were cross-sectional; whereas the eight studies in our review examining racial discrimination and BP using other designs (four RCTs and four experimental) found an association between racial discrimination and BP. Second, there were only four studies in the review that used self-reported BP, two [47,49] of which did not find an association among racial discrimination and BP. Finally, there was a clear lack of consistency among measurements of racial discrimination in studies with negative findings, with 42% of studies not specifying the measurement used.

The current findings demonstrating a link between discrimination and cardiovascular health support previous reviews on this topic. A review of 44 studies found that perceived racial discrimination was associated with hypertension, and most strongly associated with nighttime ambulatory BP, especially among AA participants [10]. Another review of 12 studies found that racism may increase the risk for hypertension and this effect is more evident for institutional racism (i.e., policies and/or procedures of institutions that result in unequal treatment for particular groups) than individual level racism (i.e., race-based mistreatment committed by individuals and targeted at other individuals) [18].

To advance this field of study, our review points to several areas in which additional research is warranted to better understand the relationship between social discrimination and cardiovascular health. In particular, it will be important for future work to employ improved methodology, including assessment of both discrimination and cardiovascular outcomes using standardized measurements and techniques consistently across studies. Increased use of objective stressors to assess cardiovascular stress responses to discrimination will be important, as will longitudinal prospective studies to assess effects of discrimination on cardiovascular health over time. In addition, studies are needed to clarify relationships between various types of discrimination and HRV/HR, BP, and blood/saliva cardiovascular biomarkers, and to identify the vulnerability to CVD among more diverse samples within stigmatized populations. Finally, the current literature consists of diverse disciplines (e.g., psychology, public health), suggesting the need for multidisciplinary/cross-disciplinary research on this issue to approach this topic from multiple perspectives.

### Strengths and limitations

Our systematic review adhered to PRISMA contemporary standards, [19] consolidating a considerable literature to examine links between commonly reported types of social discrimination and cardiovascular health indices. The comprehensive approach of this systematic review permitted the ability to identify key gaps and methodological limitations in the current literature which can inform future research studies on this topic. Although this review included prevalent types of stigmatized groups in American society, it was beyond the scope of this review to include all types of societal discrimination. More work is needed to examine cardiovascular health in the context of other types of discrimination, such as disability and religion. Furthermore, there are some topic areas of this review that include few studies; therefore, larger conclusions cannot be made for these subcategories (e.g., the association between weight discrimination and HRV/HR), indicating the need for further research examining these relationships. Also, this review only included articles published in English, and includes only studies conducted in the U.S. It is not known what differences may exist in the relationship between discrimination and cardiovascular health in different cultures where stigmatized groups (e.g., homosexuals) may be illegal resulting in heightened stress living in such a society and the implications for cardiovascular health. Cross-cultural research examining these issues will be informative in this regard. Finally, this review did not discuss the underlying mechanisms that may be responsible for the association between discrimination among stigmatized groups and adverse cardiovascular health. Although the mechanisms responsible for this association may be attributable to the way the body responds to the emotional distress of discrimination as a stressor, [11] more attention is needed to clarify underlying mechanisms that link these to increased CVD risk.

### Implications for preventive health care

In light of the consistent evidence highlighting impaired cardiovascular health among stigmatized groups, it may be informative for health care providers to assess perceived discrimination in their patients when evaluating their cardiovascular health. If patients report experiences of discrimination due to their stigmatized identity, health care providers may want to consider further evaluating patients for indicators of adverse cardiovascular health. In addition, implementing an interdisciplinary health care approach to patient care (i.e., involving health care providers from different disciplines, but coordinated toward a common and coherent approach) [120,121] could be useful to help determine if a patient’s poor cardiovascular health is linked to psychological consequences associated with perceived discrimination (e.g., stress, anxiety, and depression). For example, promoting increased communication between psychologists, primary care physicians and/or cardiologists could help facilitate the recognition and interdisciplinary treatment of patients whose health may be further compromised by discrimination. More broadly, raising awareness of the increased vulnerability for impaired cardiovascular health among stigmatized patient populations seems warranted. Health care providers may benefit from training on strategies to assess patients for experiences of discrimination, and to increase their awareness about the potential links between these experiences and cardiovascular health.

Finally, some evidence has documented the potentially harmful role of stigma in the delivery of treatment and prevention of cardiovascular disease (CVD) for individuals who are vulnerable to stigma-based inequities. For example, studies have documented implicit racial/ethnic bias by medical professionals against ethnic minorities with CVD [9,122,123], as well as lower-quality care and lower-quality clinical interactions for this patient population [124]. Considerable evidence has additionally demonstrated that medical professionals hold negative stereotypes and biases towards patients with obesity [125,126]. In response to experiences of weight stigma in the health care setting, patients with obesity are less likely to undergo health screenings and more likely to delay or avoid seeking healthcare [125,127], increasing their likelihood of having undiagnosed and untreated CVD. Thus, health care providers may themselves benefit from education about discrimination and its impact on patient health, and from broader training efforts to help reduce stigma in the health care setting that could unintentionally perpetuate adverse experiences for patients who are vulnerable to stigma and its health consequences.

## Supporting information

S1 File Full search strategy for the electronic databases queried: PubMed, PsycINFO, CINAHL, Sociological Abstracts, Academic Search Premier, Scopus (including EMBASE). (DOCX)

S1 Table All non-randomized studies were assessed for methodological quality and risk of bias using the Newcastle-Ottawa Scale (NOS). The NOS uses a ‘star’ system in which a study is assessed on three subscales including the selection of the study groups, the comparability of the groups, and the ascertainment of either the exposure or outcome of interest. The maximum score a study can receive on each of these subscales is 4, 2, and 3 ‘stars’ respectively. The highest-quality study receives 9 ‘stars.’ All randomized controlled and experimental studies included in the systematic review were assessed for study methodological quality and risk of bias using the 7-item Cochrane Collaboration tool. These criteria assessed several forms of bias including selection, performance, detection, attrition, reporting, and “other.” Studies were given a score of -1, 0, or +1 for each criterion which represented ‘high’, ‘unclear’, or ‘low’ risk, respectively.
(XLSX)

S2 Table Characteristics of studies (N = 84) examining the relationship between stigma/discrimination and cardiovascular health outcomes among common socially stigmatized groups. (DOCX)

S3 Table PRISMA checklist. (DOC)
