# Supplementary material for: Links between discrimination and cardiovascular health among socially stigmatized groups: A systematic review
Source: PLoS One. 2019 Jun 10;14(6):e0217623. doi: 10.1371/journal.pone.0217623 (PMC6557496; doi:10.1371/journal.pone.0217623)
Supplement: S1 File — (DOCX) [file pone.0217623.s001.docx]

**S1. Full search strategy for the electronic databases queried: PubMed, PsycINFO, CINAHL, Sociological Abstracts, Academic Search Premier, Scopus (including EMBASE)**

**Databases:** Databases will be searched from their inception or date of the earliest available publication.

**Inclusion Criteria:**

-Studies assessing the relationship between discrimination and cardiovascular health outcomes among stigmatized groups

-Must have assessed this relationship among one of the following stigmatized groups: gender, race/ethnicity, age, body weight/obesity, or sexual orientation

**Exclusion Criteria:**

-Non-human subjects

-Subjects aged <19 yr

-Not published

-Not published in English

-Included a stigma unrelated gender, race/ethnicity, age, body weight/obesity, or sexual orientation

-Does not contain data linking a type of discrimination among a stigmatized group to a cardiovascular health outcome

-Case studies

**PubMed (including MEDLINE)**

Vendor/Platform: National Library of Medicine

PubMed will be searched with appropriate Medical Subject Headings (MeSH) incorporated into hedges. Filters for Humans and English language are also incorporated into hedges.

**PsycINFO**

American Psychological Association

Vendor/platform: Ebscohost

Filters: English, peer review journals, humans, ages 18+

**CINAHL**

Vendor/platform: Ebscohost

Filters: English, Research Articles, Exclude MEDLINE records, human, ages 19+

**Sociological Abstracts**

Vendor/platform: Proquest

Filters: Peer reviewed, scholarly journals, English

**Academic Search Premier**

Vendor/platform: Ebscohost

Filters: Scholarly(peer reviewed) journals, articles, English

**Scopus (including EMBASE)**

Vendor/platform: Elsevier SciVerse

Filters will be set for

Document Type to include articles only.

**Key Search Terms to include:**

**Related to Stigma:**

Assumption*[ti]

attitude*[ti] (this will be included in weight search only)

attribution*[ti]

"bias"[ti]

"biased"[ti]

"biases"[ti]

blame

blaming

"bully"

"bullying"

"bullied"

"bullying"[Mesh]

discriminated[ti]

discrimination[ti]

guilt

harassment

"labeling"

perception*[ti] (this will be included in weight search only)

prejudic*

"prejudice"[Mesh]

prejudic*

racism

racism[mesh]

racist

shame

shamed

shaming

"shame"[Mesh]

"social approval"

"social discrimination"

"social discrimination"[Mesh:NoExp]

"social stigma"[Mesh]

stereotyp*

"stereotyping"[Mesh]

stigma

stigmas

stigmatiz*

stigmatis*

"Stress, Psychological/complications"[MAJR] (potentially include in weight search)

tease

teased

teasing

"treatment barrier"

"treatment barriers"

"unfair treatment"

victimiz*

**Socially Stigmatized Groups**

"African Americans/psychology"[mesh]

"African American"

"African Americans"

"Asian"

"Asians"

bisexual*

bi-sexual*

"black"

"blacks"

BMI

"body mass"

"body mass index"[Mesh]

"body weight"

"body weight"[Mesh]

"Chinese"

ethnic*

ethnology[sh]

fat

fatness

female*

gay [tiab]

gender

(heavy[ti] AND weight[ti])

"Hispanic"

"Hispanics*

homosexual*

"Japanese"

minorit*

obese

obesity

"obesity"[Mesh]

overweight

"over weight"

"overweight"[Mesh]

"physical appearance"

"physical appearances"

"race"

"racial"

racism

racism[mesh]

racist

"sexual orientation"

sexuality

transsexual*

"queer"

weight[ti]

"women"

**Related to Cardiovascular health:**

1. **Blood pressure**

"blood pressure"

"blood pressures"

"blood pressure"[Mesh:NoExp]

hypertensi*

"hypertension"[Mesh]

b) **Heart rate**

"heart rate"

"heart rate"[Mesh]

pulse[ti]

"pulse"[Mesh]

"pulse rate"

1. **Cardiac Biomarkers**

Amylase

Atherosclerosis

"Atherosclerosis"[Mesh]

"Carotid Intima-Media Thickness"[Mesh]

Cardiometabolic

Cholesterol

Cortisol

Glucose

impedance

"Impedance cardiography"

Insulin

Interleukin

"intima-media thickness"

“intimal medial thickness”

Lipoprotein*

Triglyceride*

1. **Other**

aneurysm*

"aneurysm"[Mesh]

"aortic disease"

"aortic diseases"

"aortic diseases"[Mesh]

atherosclerosis

"cardiovascular biomarker"

"cardiovascular biomarkers"

"cardiovascular disease"

"cardiovascular diseases"

"cardiovascular diseases"[Mesh]

"cardiovascular diseases/ethnology"[MAJR]

"CVD"

"heart arrest"

"heart attack"

"heart attacks"

"heart disease"

"heart diseases"

"heart failure"

"myocardial infarct"

"myocardial infarction"

"myocardial infarctions"

"myocardial infarction"[Mesh]

"myocardial ischemia"

stroke

"stroke"[Mesh]

"vascular disease"

"vascular diseases"

"vascular diseases"[Mesh]

***The Link Between Discrimination and Blood Pressure Among Socially Stigmatized Groups***

**PubMed:**

PubMed search 1:

(assumption*[ti] OR attribution*[ti] OR "bias"[ti] OR "biased"[ti] OR "biases"[ti] OR blame OR blaming OR "bully" OR "bullying" OR "bullied" OR "bullying"[Mesh] OR discriminated[ti] OR discrimination[ti] OR guilt OR harassment OR "labeling" OR prejudic* OR "prejudice"[Mesh] OR racism OR racism[mesh] OR racist OR shame OR shamed OR shaming OR "shame"[Mesh] OR "social approval" OR "social discrimination" OR "social discrimination"[Mesh:NoExp] OR "social stigma"[Mesh] OR stereotyp* OR "stereotyping"[Mesh] OR stigma OR stigmas OR stigmatiz* OR stigmatis* OR tease OR teased OR teasing OR "treatment barrier" OR "treatment barriers" OR "unfair treatment" OR victimiz*) AND ("African Americans/psychology"[mesh] OR "African American"[tiab] OR "African Americans" OR "Asian"[tiab] OR "Asians"[tiab] OR bisexual* OR bi-sexual* OR "black"[tiab] OR "blacks" OR BMI OR "body mass" OR "body mass index"[Mesh] OR "body weight" OR "body weight"[Mesh] OR "Chinese"[tiab] OR ethnic* OR ethnology[sh] OR fat OR fatness OR female*[tiab] OR gay [tiab] OR gender[tiab] OR (heavy[ti] AND weight[ti]) OR "Hispanic"[tiab] OR Hispanics[tiab] OR homosexual* OR "Japanese"[tiab] OR minorit*[tiab] OR obese OR obesity[tiab] OR "obesity"[Mesh] OR overweight OR "over weight" OR "overweight"[Mesh] OR "physical appearance" OR "physical appearances" OR "race" OR racial[tiab] OR racism OR racism[mesh] OR racist OR "sexual orientation" OR sexuality OR transsexual* OR "queer" OR weight[ti] OR women[tiab]) AND ("blood pressure" OR "blood pressures" OR "blood pressure"[Mesh:NoExp] OR hypertensi* OR "hypertension"[Mesh]) AND "adult"[mesh] AND English[la] NOT ("birth weight"[ti] OR "fetal weight" OR "fat soluble" OR "recall bias" OR "Discrimination Learning"[Mesh] OR "pharmacokinetics"[sh] OR "Tomography, X-Ray"[Mesh] OR "Weight Lifting"[Mesh] OR "weight bearing" OR "Selection Bias"[Mesh] OR "food labeling" OR "spin labeling" OR "Staining and Labeling"[MeSH] OR "taste perception" OR salt[ti] OR sodium[ti] OR "Mutation"[mesh] OR "Genotype"[mesh] OR "Alleles"[mesh] OR "Genetic Association Studies"[mesh] OR telomere* OR "Bias (Epidemiology)"[mesh] OR isotope* OR "labeling guidelines" OR mice[mesh] OR "movement/physiology"[mesh] OR erythrocyte*[ti] OR "Nick-End Labeling" OR "spatial labeling" OR "pulse labeling" OR discriminators[ti] OR "discrimination ability" OR "superior discrimination" OR "improve discrimination" OR "improved discrimination" OR "validation studies"[mesh] OR comment[pt] OR editorial[pt])

PubMed search 2:

(sexism[ti] OR sexist[ti] OR racist[ti] OR racism[ti] OR stigma[ti] OR stigmas[ti] OR stimatiz*[ti] OR (race[ti] AND discrim*[ti]) OR (racial[ti] AND discrim*[ti])) AND ("blood pressure" OR "blood pressures" OR "blood pressure"[Mesh:NoExp] OR hypertensi* OR "hypertension"[Mesh])

**Other databases:**

Search 1:

Line 1 (in title): assumption* OR attribution* OR "bias" OR "biased" OR "biases" OR discriminated OR discrimination OR "labeling" OR prejudic*

OR

Line 2: blame OR blaming OR "bully" OR "bullying" OR "bullied" OR guilt OR harassment OR racism OR racist OR shame OR shamed OR shaming OR "social approval" OR "social discrimination" OR stereotyp* OR stigma OR stigmas OR stigmatiz* OR stigmatis* OR tease OR teased OR teasing OR "treatment barrier" OR "treatment barriers" OR "unfair treatment" OR victimiz*

AND Search 2:

Line 1 (in title): weight

OR

Line 2: "African American" OR "African Americans" OR "Asian" OR "Asians" OR bisexual* OR bi-sexual* OR "black" OR "blacks" OR BMI OR "body mass" OR "body weight" OR "Chinese" OR ethnic* OR fat OR fatness OR female* OR gay OR gender OR "Hispanic" OR Hispanics OR homosexual* OR "Japanese" OR minorit* OR obese OR obesity OR overweight OR "over weight" OR "physical appearance" OR "physical appearances" OR "race" OR racial OR racism OR racist OR "sexual orientation" OR sexuality OR transsexual* OR "queer" OR women

AND Search 3:

Line 1: "blood pressure" OR "blood pressures" OR hypertensi*

NOT Search 4:

Line 1 (in title): "birth weight" OR discriminators OR sodium OR salt OR mutation* OR Genotype* OR Allele* OR "Genetic Association Study" OR GWAS OR mice OR erythrocyte* OR comment* OR editorial OR letter

OR

Line 2: "fetal weight" OR "fat soluble" OR "recall bias" OR "Discrimination Learning" OR "pharmacokinetics" OR "weight lifting" OR "weight bearing" OR "Selection Bias" OR "food labeling" OR "spin labeling" OR "Staining and Labeling" OR "taste perception" OR telomere* OR isotope* OR "labeling guidelines" OR "spatial labeling" OR "pulse labeling" OR "Nick-End Labeling" OR "discrimination ability" OR "superior discrimination" OR "improve discrimination" OR "improved discrimination"

***The Link Between Discrimination and Heart Rate/HRV Among Socially Stigmatized Groups***

**PubMed:**

(assumption*[ti] OR attribution*[ti] OR "bias"[ti] OR "biased"[ti] OR "biases"[ti] OR blame OR blaming OR "bully" OR "bullying" OR "bullied" OR "bullying"[Mesh] OR discriminated[ti] OR discrimination[ti] OR guilt OR harassment OR "labeling" OR prejudic* OR "prejudice"[Mesh] OR racism OR racism[mesh] OR racist OR shame OR shamed OR shaming OR "shame"[Mesh] OR "social approval" OR "social discrimination" OR "social discrimination"[Mesh:NoExp] OR "social stigma"[Mesh] OR stereotyp* OR "stereotyping"[Mesh] OR stigma OR stigmas OR stigmatiz* OR stigmatis* OR tease OR teased OR teasing OR "treatment barrier" OR "treatment barriers" OR "unfair treatment" OR victimiz*) AND ("African Americans/psychology"[mesh] OR "African American"[tiab] OR "African Americans" OR "Asian"[tiab] OR "Asians"[tiab] OR bisexual* OR bi-sexual* OR "black"[tiab] OR "blacks" OR BMI OR "body mass" OR "body mass index"[Mesh] OR "body weight" OR "body weight"[Mesh] OR "Chinese"[tiab] OR ethnic* OR ethnology[sh] OR fat OR fatness OR female*[tiab] OR gay [tiab] OR gender[tiab] OR (heavy[ti] AND weight[ti]) OR "Hispanic"[tiab] OR Hispanics[tiab] OR homosexual* OR "Japanese"[tiab] OR minorit*[tiab] OR obese OR obesity[tiab] OR "obesity"[Mesh] OR overweight OR "over weight" OR "overweight"[Mesh] OR "physical appearance" OR "physical appearances" OR "race" OR racial[tiab] OR racism OR racism[mesh] OR racist OR "sexual orientation" OR sexuality OR transsexual* OR "queer" OR weight[ti] OR women[tiab]) AND ("heart rate" OR "heart rate"[Mesh] OR pulse[ti] OR "pulse"[Mesh] OR "pulse rate") AND "adult"[mesh] AND English[la] NOT ("birth weight"[ti] OR "fetal weight" OR "fat soluble" OR "recall bias" OR "Discrimination Learning"[Mesh] OR "pharmacokinetics"[sh] OR "Tomography, X-Ray"[Mesh] OR "Weight Lifting"[Mesh] OR "weight bearing" OR "Selection Bias"[Mesh] OR "food labeling" OR "spin labeling" OR "Staining and Labeling"[MeSH] OR "taste perception" OR salt[ti] OR sodium[ti] OR "Mutation"[mesh] OR "Genotype"[mesh] OR "Alleles"[mesh] OR "Genetic Association Studies"[mesh] OR telomere* OR "Bias (Epidemiology)"[mesh] OR isotope* OR "labeling guidelines" OR mice[mesh] OR "movement/physiology"[mesh] OR erythrocyte*[ti] OR "Nick-End Labeling" OR "spatial labeling" OR "pulse labeling" OR discriminators[ti] OR "discrimination ability" OR "validation studies"[mesh] OR comment[pt] OR editorial[pt])

PubMed search 2:

(sexism[ti] OR sexist[ti] OR racist[ti] OR racism[ti] OR stigma[ti] OR stigmas[ti] OR stimatiz*[ti] OR ("race"[ti] AND discrim*[ti]) OR ("racial"[ti] AND discrim*[ti])) AND ("heart rate" OR pulse)

**Other databases:**

Search 1:

Line 1 (in title): assumption* OR attribution* OR "bias" OR "biased" OR "biases" OR discriminated OR discrimination OR "labeling" OR prejudic*

OR

Line 2: blame OR blaming OR "bully" OR "bullying" OR "bullied" OR guilt OR harassment OR racism OR racist OR shame OR shamed OR shaming OR "social approval" OR "social discrimination" OR stereotyp* OR stigma OR stigmas OR stigmatiz* OR stigmatis* OR tease OR teased OR teasing OR "treatment barrier" OR "treatment barriers" OR "unfair treatment" OR victimiz*

AND Search 2:

Line 1 (in title): weight

OR

Line 2: "African American" OR "African Americans" OR "Asian" OR "Asians" OR bisexual* OR bi-sexual* OR "black" OR "blacks" OR BMI OR "body mass" OR "body weight" OR "Chinese" OR ethnic* OR fat OR fatness OR female* OR gay OR gender OR "Hispanic" OR Hispanics OR homosexual* OR "Japanese" OR minorit* OR obese OR obesity OR overweight OR "over weight" OR "physical appearance" OR "physical appearances" OR "race" OR racial OR racism OR racist OR "sexual orientation" OR sexuality OR transsexual* OR "queer" OR women

AND Search 3:

Line 1: "heart rate" OR "pulse rate"

OR

Line 2 (in title): pulse

NOT Search 4:

Line 1 (in title): "birth weight" OR discriminators OR sodium OR salt OR mutation* OR Genotype* OR Allele* OR "Genetic Association Study" OR GWAS OR mice OR erythrocyte* OR comment* OR editorial OR letter

OR

Line 2: "fetal weight" OR "fat soluble" OR "recall bias" OR "Discrimination Learning" OR "pharmacokinetics" OR "weight lifting" OR "weight bearing" OR "Selection Bias" OR "food labeling" OR "spin labeling" OR "Staining and Labeling" OR "taste perception" OR telomere* OR isotope* OR "labeling guidelines" OR "spatial labeling" OR "pulse labeling" OR "Nick-End Labeling" OR "discrimination ability" OR "superior discrimination" OR "improve discrimination" OR "improved discrimination"

***The Link Between Discrimination and Cardiovascular Biomarkers Among Socially Stigmatized Groups***

PubMed:

(assumption*[ti] OR attribution*[ti] OR "bias"[ti] OR "biased"[ti] OR "biases"[ti] OR blame OR blaming OR "bully" OR "bullying" OR "bullied" OR "bullying"[Mesh] OR discriminated[ti] OR discrimination[ti] OR guilt OR harassment OR "labeling"[ti] OR prejudic*[ti] OR "prejudice"[Mesh] OR racism OR racism[mesh] OR racist OR shame OR shamed OR shaming OR "shame"[Mesh] OR "social approval" OR "social discrimination" OR "social discrimination"[Mesh:NoExp] OR "social stigma"[Mesh] OR stereotyp* OR "stereotyping"[Mesh] OR stigma OR stigmas OR stigmatiz* OR stigmatis* OR tease OR teased OR teasing OR "treatment barrier" OR "treatment barriers" OR "unfair treatment" OR victimiz*) AND ("African Americans/psychology"[mesh] OR "African American"[tiab] OR "African Americans" OR "Asian"[tiab] OR "Asians"[tiab] OR bisexual* OR bi-sexual* OR "black"[tiab] OR "blacks" OR BMI OR "body mass" OR "body mass index"[Mesh] OR "body weight" OR "body weight"[Mesh] OR "Chinese"[tiab] OR ethnic* OR ethnology[sh] OR fat OR fatness OR female*[tiab] OR gay [tiab] OR gender[tiab] OR (heavy[ti] AND weight[ti]) OR "Hispanic"[tiab] OR Hispanics[tiab] OR homosexual* OR "Japanese"[tiab] OR minorit*[tiab] OR obese OR obesity[tiab] OR "obesity"[Mesh] OR overweight OR "over weight" OR "overweight"[Mesh] OR "physical appearance" OR "physical appearances" OR "race" OR racial[tiab] OR racism OR racism[mesh] OR racist OR "sexual orientation" OR sexuality OR transsexual* OR "queer" OR weight[ti] OR women[tiab]) AND (amylase OR atherosclerosis[tiab] OR "atherosclerosis"[Mesh] OR "carotid intima-media thickness"[Mesh] OR cardiometabolic OR cholesterol OR cortisol OR glucose OR impedance OR "impedance cardiography" OR insulin OR interleukin OR lipoprotein* OR triglyceride*) AND "adult"[mesh] AND English[la] NOT ("birth weight"[ti] OR "fetal weight" OR "fat soluble" OR "recall bias" OR "Discrimination Learning"[Mesh] OR "pharmacokinetics"[sh] OR "Tomography, X-Ray"[Mesh] OR "Weight Lifting"[Mesh] OR "weight bearing" OR "Selection Bias"[Mesh] OR "food labeling" OR "spin labeling" OR "Staining and Labeling"[MeSH] OR "taste perception" OR salt[ti] OR sodium[ti] OR "Mutation"[mesh] OR "Genotype"[mesh] OR "Alleles"[mesh] OR "Genetic Association Studies"[mesh] OR telomere* OR "Bias (Epidemiology)"[mesh] OR isotope* OR "labeling guidelines" OR mice[mesh] OR "movement/physiology"[mesh] OR erythrocyte*[ti] OR "Nick-End Labeling" OR "spatial labeling" OR "pulse labeling" OR discriminators[ti] OR "discrimination ability" OR "superior discrimination" OR "improve discrimination" OR "improved discrimination" OR "validation studies"[mesh] OR comment[pt] OR editorial[pt])

PubMed search 2:

(sexism[ti] OR sexist[ti] OR racist[ti] OR racism[ti] OR stigma[ti] OR stigmas[ti] OR stimatiz*[ti] OR ("race"[ti] AND discrim*[ti]) OR ("racial"[ti] AND discrim*[ti])) AND (amylase OR atherosclerosis[tiab] OR "atherosclerosis"[Mesh] OR "carotid intima-media thickness"[Mesh] OR cardiometabolic OR cholesterol OR cortisol OR glucose OR impedance OR "impedance cardiography" OR insulin OR interleukin OR lipoprotein* OR triglyceride*) NOT ("birth weight"[ti] OR "fetal weight" OR "fat soluble" OR "recall bias" OR "Discrimination Learning"[Mesh] OR "pharmacokinetics"[sh] OR "Tomography, X-Ray"[Mesh] OR "Weight Lifting"[Mesh] OR "weight bearing" OR "Selection Bias"[Mesh] OR "food labeling" OR "spin labeling" OR "Staining and Labeling"[MeSH] OR "taste perception" OR salt[ti] OR sodium[ti] OR "Mutation"[mesh] OR "Genotype"[mesh] OR "Alleles"[mesh] OR "Genetic Association Studies"[mesh] OR telomere* OR "Bias (Epidemiology)"[mesh] OR isotope* OR "labeling guidelines" OR mice[mesh] OR "movement/physiology"[mesh] OR erythrocyte*[ti] OR "Nick-End Labeling" OR "spatial labeling" OR "pulse labeling" OR discriminators[ti] OR "discrimination ability" OR "superior discrimination" OR "improve discrimination" OR "improved discrimination" OR "validation studies"[mesh] OR comment[pt] OR editorial[pt])

Other databases:

Search 1:

Line 1 (in title): assumption* OR attribution* OR "bias" OR "biased" OR "biases" OR discriminated OR discrimination OR "labeling" OR prejudic*

OR

Line 2: blame OR blaming OR "bully" OR "bullying" OR "bullied" OR guilt OR harassment OR racism OR racist OR shame OR shamed OR shaming OR "social approval" OR "social discrimination" OR stereotyp* OR stigma OR stigmas OR stigmatiz* OR stigmatis* OR tease OR teased OR teasing OR "treatment barrier" OR "treatment barriers" OR "unfair treatment" OR victimiz*

AND Search 2:

Line 1 (in title): weight

OR

Line 2: "African American" OR "African Americans" OR "Asian" OR "Asians" OR bisexual* OR bi-sexual* OR "black" OR "blacks" OR BMI OR "body mass" OR "body weight" OR "Chinese" OR ethnic* OR fat OR fatness OR female* OR gay OR gender OR "Hispanic" OR Hispanics OR homosexual* OR "Japanese" OR minorit* OR obese OR obesity OR overweight OR "over weight" OR "physical appearance" OR "physical appearances" OR "race" OR racial OR racism OR racist OR "sexual orientation" OR sexuality OR transsexual* OR "queer" OR women

AND Search 3:

Line 1: amylase OR atherosclerosis OR "atherosclerosis"[Mesh] OR "carotid intima-media thickness" OR cardiometabolic OR cholesterol OR cortisol OR glucose OR impedance OR "impedance cardiography" OR insulin OR interleukin OR "intramedial thickness" OR lipoprotein* OR triglyceride*

NOT Search 4:

Line 1 (in title): "birth weight" OR discriminators OR sodium OR salt OR mutation* OR Genotype* OR Allele* OR "Genetic Association Study" OR GWAS OR mice OR erythrocyte* OR comment* OR editorial OR letter

OR

Line 2: "fetal weight" OR "fat soluble" OR "recall bias" OR "Discrimination Learning" OR "pharmacokinetics" OR "weight lifting" OR "weight bearing" OR "Selection Bias" OR "food labeling" OR "spin labeling" OR "Staining and Labeling" OR "taste perception" OR telomere* OR isotope* OR "labeling guidelines" OR "spatial labeling" OR "pulse labeling" OR "Nick-End Labeling" OR "discrimination ability" OR "superior discrimination" OR "improve discrimination" OR "improved discrimination"

***The Link Between Discrimination and “Other” Among Socially Stigmatized Groups***

PubMed:

PubMed Search 1:

(assumption*[ti] OR attribution*[ti] OR "bias"[ti] OR "biased"[ti] OR "biases"[ti] OR blame OR blaming OR "bully" OR "bullying" OR "bullied" OR "bullying"[Mesh] OR discriminated[ti] OR discrimination[ti] OR guilt OR harassment OR "labeling" OR prejudic*[ti] OR "prejudice"[Mesh] OR racism OR racism[mesh] OR racist OR shame OR shamed OR shaming OR "shame"[Mesh] OR "social approval" OR "social discrimination" OR "social discrimination"[Mesh:NoExp] OR "social stigma"[Mesh] OR stereotyp* OR "stereotyping"[Mesh] OR stigma OR stigmas OR stigmatiz* OR stigmatis* OR tease OR teased OR teasing OR "treatment barrier" OR "treatment barriers" OR "unfair treatment" OR victimiz*) AND ("African Americans/psychology"[mesh] OR "African American"[tiab] OR "African Americans" OR "Asian"[tiab] OR "Asians"[tiab] OR bisexual* OR bi-sexual* OR "black"[tiab] OR "blacks" OR BMI OR "body mass" OR "body mass index"[Mesh] OR "body weight" OR "body weight"[Mesh] OR "Chinese"[tiab] OR ethnic* OR ethnology[sh] OR fat OR fatness OR female*[tiab] OR gay [tiab] OR gender[tiab] OR (heavy[ti] AND weight[ti]) OR "Hispanic"[tiab] OR Hispanics[tiab] OR homosexual* OR "Japanese"[tiab] OR minorit*[tiab] OR obese OR obesity[tiab] OR "obesity"[Mesh] OR overweight OR "over weight" OR "overweight"[Mesh] OR "physical appearance" OR "physical appearances" OR "race" OR racial[tiab] OR racism OR racism[mesh] OR racist OR "sexual orientation" OR sexuality OR transsexual* OR "queer" OR weight[ti] OR women[tiab]) AND (aneurysm* OR "aneurysm"[Mesh] OR "aortic disease" OR "aortic diseases" OR "aortic diseases"[Mesh] OR atherosclerosis OR "cardiovascular biomarker" OR "cardiovascular biomarkers" OR "cardiovascular disease" OR "cardiovascular diseases" OR "cardiovascular diseases"[Mesh] OR "CVD" OR "heart arrest" OR "heart attack" OR "heart attacks" OR "heart disease"[tiab] OR "heart diseases" OR "heart failure" OR "myocardial infarct" OR "myocardial infarction" OR "myocardial infarctions" OR "myocardial infarction"[Mesh] OR "myocardial ischemia" OR "stroke"[tiab] OR "stroke"[Mesh] OR "vascular disease" OR "vascular diseases" OR "vascular diseases"[Mesh]) AND "adult"[mesh] AND English[la] NOT ("birth weight"[ti] OR "fetal weight" OR "fat soluble" OR "recall bias" OR "Discrimination Learning"[Mesh] OR "pharmacokinetics"[sh] OR "Tomography, X-Ray"[Mesh] OR "Weight Lifting"[Mesh] OR "weight bearing" OR "Selection Bias"[Mesh] OR "food labeling" OR "spin labeling" OR "Staining and Labeling"[MeSH] OR "taste perception" OR salt[ti] OR sodium[ti] OR "Mutation"[mesh] OR "Genotype"[mesh] OR "Alleles"[mesh] OR "Genetic Association Studies"[mesh] OR telomere* OR "Bias (Epidemiology)"[mesh] OR isotope* OR "labeling guidelines" OR "spatial labeling" OR "pulse labeling" OR discriminators[ti] OR "discrimination ability" OR "superior discrimination" OR "improve discrimination" OR "improved discrimination" OR mice[mesh] OR "movement/physiology"[mesh] OR erythrocyte*[ti] OR "Nick-End Labeling" OR "validation studies"[mesh] OR comment[pt] OR editorial[pt])

PubMed search 2:

(sexism[ti] OR sexist[ti] OR racist[ti] OR racism[ti] OR stigma[ti] OR stigmas[ti] OR stimatiz*[ti] OR ("race"[ti] AND discrim*[ti]) OR ("racial"[ti] AND discrim*[ti])) AND (aneurysm* OR "aneurysm"[Mesh] OR "aortic disease" OR "aortic diseases" OR "aortic diseases"[Mesh] OR atherosclerosis OR "cardiovascular biomarker" OR "cardiovascular biomarkers" OR "cardiovascular disease" OR "cardiovascular diseases" OR "cardiovascular diseases"[Mesh] OR "CVD" OR "heart arrest" OR "heart attack" OR "heart attacks" OR "heart disease"[tiab] OR "heart diseases" OR "heart failure" OR "myocardial infarct" OR "myocardial infarction" OR "myocardial infarctions" OR "myocardial infarction"[Mesh] OR "myocardial ischemia" OR "stroke"[tiab] OR "stroke"[Mesh] OR "vascular disease" OR "vascular diseases" OR "vascular diseases"[Mesh]) NOT ("birth weight"[ti] OR "fetal weight" OR "fat soluble" OR "recall bias" OR "Discrimination Learning"[Mesh] OR "pharmacokinetics"[sh] OR "Tomography, X-Ray"[Mesh] OR "Weight Lifting"[Mesh] OR "weight bearing" OR "Selection Bias"[Mesh] OR "food labeling" OR "spin labeling" OR "Staining and Labeling"[MeSH] OR "taste perception" OR salt[ti] OR sodium[ti] OR "Mutation"[mesh] OR "Genotype"[mesh] OR "Alleles"[mesh] OR "Genetic Association Studies"[mesh] OR telomere* OR "Bias (Epidemiology)"[mesh] OR isotope* OR "labeling guidelines" OR "spatial labeling" OR "pulse labeling" OR discriminators[ti] OR "discrimination ability" OR "superior discrimination" OR "improve discrimination" OR "improved discrimination" OR mice[mesh] OR "movement/physiology"[mesh] OR erythrocyte*[ti] OR "Nick-End Labeling" OR "validation studies"[mesh] OR comment[pt] OR editorial[pt])

Other databases:

Search 1:

Line 1 (in title): assumption* OR attribution* OR "bias" OR "biased" OR "biases" OR discriminated OR discrimination OR "labeling" OR prejudic*

OR

Line 2: blame OR blaming OR "bully" OR "bullying" OR "bullied" OR guilt OR harassment OR racism OR racist OR shame OR shamed OR shaming OR "social approval" OR "social discrimination" OR stereotyp* OR stigma OR stigmas OR stigmatiz* OR stigmatis* OR tease OR teased OR teasing OR "treatment barrier" OR "treatment barriers" OR "unfair treatment" OR victimiz*

AND Search 2:

Line 1 (in title): weight

OR

Line 2: "African American" OR "African Americans" OR "Asian" OR "Asians" OR bisexual* OR bi-sexual* OR "black" OR "blacks" OR BMI OR "body mass" OR "body weight" OR "Chinese" OR ethnic* OR fat OR fatness OR female* OR gay OR gender OR "Hispanic" OR Hispanics OR homosexual* OR "Japanese" OR minorit* OR obese OR obesity OR overweight OR "over weight" OR "physical appearance" OR "physical appearances" OR "race" OR racial OR racism OR racist OR "sexual orientation" OR sexuality OR transsexual* OR "queer" OR women

AND Search 3:

Line 1: aneurysm* OR "aortic disease" OR "aortic diseases" OR atherosclerosis OR "cardiovascular biomarker" OR "cardiovascular biomarkers" OR "cardiovascular disease" OR "cardiovascular diseases" OR "CVD" OR "heart arrest" OR "heart attack" OR "heart attacks" OR "heart disease" OR "heart diseases" OR "heart failure" OR "myocardial infarct" OR "myocardial infarction" OR "myocardial infarctions" OR "myocardial ischemia" OR stroke OR "vascular disease" OR "vascular diseases"

NOT Search 4:

Line 1 (in title): "birth weight" OR discriminators OR sodium OR salt OR mutation* OR Genotype* OR Allele* OR "Genetic Association Study" OR GWAS OR mice OR erythrocyte* OR comment* OR editorial OR letter

OR

Line 2: "fetal weight" OR "fat soluble" OR "recall bias" OR "Discrimination Learning" OR "pharmacokinetics" OR "weight lifting" OR "weight bearing" OR "Selection Bias" OR "food labeling" OR "spin labeling" OR "Staining and Labeling" OR "taste perception" OR telomere* OR isotope* OR "labeling guidelines" OR "spatial labeling" OR "pulse labeling" OR "Nick-End Labeling" OR "discrimination ability" OR "superior discrimination" OR "improve discrimination" OR "improved discrimination"
