# Supplementary material for: Links between discrimination and cardiovascular health among socially stigmatized groups: A systematic review
Source: PLoS One. 2019 Jun 10;14(6):e0217623. doi: 10.1371/journal.pone.0217623 (PMC6557496; doi:10.1371/journal.pone.0217623)
Supplement: S2 Table — (DOCX) [file pone.0217623.s003.docx]

| S2 Table. Characteristics of studies (N=84) examining the relationship between stigma/discrimination and cardiovascular health outcomes among common socially stigmatized groups. | | | | | | | | | | | | | | | | | | | | | | | | | | | | | | | | | | | | | | | | | | | | | | | | | | | | | |  |
| --- | --- | --- | --- | --- | --- | --- | --- | --- | --- | --- | --- | --- | --- | --- | --- | --- | --- | --- | --- | --- | --- | --- | --- | --- | --- | --- | --- | --- | --- | --- | --- | --- | --- | --- | --- | --- | --- | --- | --- | --- | --- | --- | --- | --- | --- | --- | --- | --- | --- | --- | --- | --- | --- | --- |
| **Author**  **(year)** | | **Study Design** | | | | | | | | | **Study Population** | | | | | | | | | | **Measure of Stigma and/or Discrimination** | | | | | | **Measure of Cardiovascular Health Indices** | | | | | | | **Length of Study** | | | | | | | | | **Study Findings*** | | | | | | | | **Significant relationship between stigma/ discrimination and cardiovascular health** | | | |
| **Blood Pressure as Primary Cardiovascular Health Outcome (n=45)** | | | | | | | | | | | | | | | | | | | | | | | | | | | | | | | | | | | | | | | | | | | | | | | | | | | | | | |
| ***Race*** | | | | | | | | | | | | | | | | | | | | | | | | | | | | | | | | | | | | | | | | | | | | | | | | | | | | | | |
| Thayer et al.  (2017) | | Cross-sectional | | | | | | | | | American Indian men (n=21) and women (n=56) aged 41.3±9.4 years from the Healing Hearts study; SBP=127.2±14.0 mmHg, DBP=77.4±9.4 mmHg | | | | | | | | | | | Racial discrimination assessed using the Williams Everyday Discrimination Scale | | | | | Resting BP was measured using a standard mercury sphygmanometer according to the JNC-6 guidelines | | | | | | | 3 days | | | | | | | | | Racial discrimination was associated with higher DBP and trended with higher SBP. | | | | | | | | Yes | | | |
| Beatty Moody et al.  (2016) | | Cross-sectional | | | | | | | | | Black (n=318) and Latino (n=289), men (n=309) women (n=298) aged 39.1±9.5 years from the Racism, Coping, and 24-hr Ambulatory BP study; Daytime ambulatory SBP=133.1±15.5 mmHg, DBP=79.5±9.8 mmHg | | | | | | | | | | | Lifetime exposure to racism/ethnic discrimination assessed using the Perceived Ethnic Discrimination Questionnaire-Community Version | | | | | BP was measured using ambulatory BP monitoring across a 24-hr period. | | | | | | | 3 visits within 2 weeks | | | | | | | | | Exposure to racial/ethnic discrimination across the lifespan was associated with elevated ambulatory SBP across the entire sample, and age moderated the association between discrimination and 24-hr and daytime DBP. | | | | | | | | Yes | | | |
| Orom et al.  (2016) | | Cross-sectional | | | | | | | | | Black (n=190), Caucasian (n=1193), Hispanic (n=120) and other (n=30) men aged 63.2±7.9 years diagnosed with clinically localized prostate cancer; SBP=134.3±15.7 mmHg, DBP=79.5±9.9 mmHg | | | | | | | | | | | Lifetime racial/ethnic discrimination assessed using the Experiences of Discrimination scale; Stigma consciousness using the Stigma Consciousness Questionnaire | | | | | Self-reported diagnosis of HTN and clinic-assessed resting BP abstracted from participants’ clinic notes | | | | | | | 1 visit | | | | | | | | | Minorities reported more racial discrimination and stigma consciousness than Whites. Discrimination and stigma consciousness was associated with higher DBP and greater odds of having HTN, respectively. | | | | | | | | Yes | | | |
| Dawson et al.  (2015) | | Cross-sectional | | | | | | | | | Black (n=371) and White (n=231), men (n=369) and women (n=233), aged 61.5±10.9, with type 2 diabetes; SBP=129.7 mmHg, DBP not examined | | | | | | | | | | | Previously validated items from the Diabetes Study of North California | | | | | SBP and hemoglobin A1c were abstracted from electronic medical records using values within 6 months | | | | | | | 1 visit | | | | | | | | | AAs perceived discrimination was significantly associated with higher SBP compared to Caucasians. | | | | | | | | Yes | | | |
| Wagner et al.  (2015) | | Cross-sectional | | | | | | | | | Black (n=39) and White (n=38) women aged 55.7±11.8 years with type 2 diabetes; Ambulatory awake SBP=124.7±13.8 mmHg, DBP=73.4±8.7 mmHg | | | | | | | | | | | Lifetime exposure to racial discrimination assessed using the Schedule of Racist Events | | | | | BP measured using 24-hr ambulatory BP monitors according to guidelines by the American Society of Hypertension | | | | | | | 1 visit | | | | | | | | | Racial discrimination was associated with higher levels of awake SBP and DBP, as well as sleep SBP and DBP in women with diabetes who are low in neuroticism. | | | | | | | | Yes | | | |
| Gregoski et al.  (2013) | | Cross-sectional | | | | | | | | | AA (n=175, ambulatory awake SBP=120.5±9.5 mmHg, DBP=72.6±7.7 mmHg) and European American (n=177) men (n=162, Ambulatory awake SBP=119.8±9.9 mmHg, DBP=71.5±6.6 mmHg) and women (n=190) aged 18.3± 2.7 years | | | | | | | | | | | Perceived lifetime discrimination assessed using the Everyday Discrimination Scale | | | | | BP measured using 24-hr ambulatory BP monitors according to the European Society of Hypertension Working Group on BP Monitoring | | | | | | | Data from 1 visit | | | | | | | | | AA ET-1 carriers with higher everyday discrimination levels had increases in nighttime DBP, and reduced nocturnal SBP and DBP dipping. | | | | | | | | Yes | | | |
| Krieger et al.  (2013) | | Cross-sectional | | | | | | | | | Black (n=504, 63% with HTN) and White (n=501, 41% with HTN) men (n=340) and women (n=665) aged 49 years | | | | | | | | | | | Lifetime and recent discrimination assessed using the Experiences of Discrimination and the Everyday Discrimination Scale; Unconscious associations using the Implicit Association Test; Structural discrimination using Jim Crow birthplace status | | | | | Resting BP measured using an automatic BP monitor following a 3-minute relaxation video | | | | | | | 1 visit | | | | | | | | | Despite worse discrimination exposure among AA participants, racial discrimination and socioeconomic position were not associated with the risk of HTN (i.e., SBP > 140 and/or DBP >90 mmHg, and/or on antihypertensive medication) or a higher Framingham CVD risk score. | | | | | | | | No | | | |
| Chae et al.  (2012) | | Cross-sectional | | | | | | | | | AA men (n=91, 46% with HTN) aged 43.9±5.8 years from the Bay Area Heart Health Study | | | | | | | | | | | Racial discrimination assessed using the Everyday Discrimination Scale; Implicit racial bias using the Black-White Implicit Association Test | | | | | Resting BP measured using an automatic BP monitor | | | | | | | 1 visit | | | | | | | | | No main effects for implicit racial bias were found. Sub-analysis found that among participants with implicit anti-black bias, more frequent reports of discrimination were associated with a higher probability of HTN (i.e., SBP > 140 and/or DBP >90 mmHg, and/or on antihypertensive medication). | | | | | | | | No, main analysis  Yes, sub-analysis | | | |
| Kaholokula et al.  (2012) | | Cross-sectional | | | | | | | | | Native Hawaiian men (n=42) and women (n=104) aged >18 years; SBP=130.4±7.4 mmHg, DBP=78.3±11.1 mmHg | | | | | | | | | | | Perceived racism assessed using shortened version of Oppression Questionnaire | | | | | Seated BP taken twice with 1 minute between using mercury sphygmomanometer on the right arm | | | | | | | 1 visit | | | | | | | | | Higher self-reported racism associated with higher SBP. | | | | | | | | Yes | | | |
| Mujahid et al.  (2011) | | Cross-sectional | | | | | | | | | AA (n=1,159), Hispanic (n=415), and Caucasian (n=1,105) men (n= 1,236) and women (n=1,443) aged 45-84 years, and 49% with HTN, from MESA | | | | | | | | | | | Chronic burden, perceived discrimination, and everyday discrimination assessed using the chronic burden scale, a perceived discrimination scaled from the Detroit Area Study, and an everyday discrimination scale also adapted from the Detroit Area Study, respectively | | | | | Resting seated BP measured three times using an automated sphygmomanometer | | | | | | | 1 visit | | | | | | | | | Prevalence of HTN (i.e., SBP > 140 and/or DBP >90 mmHg, and/or on antihypertensive medication) was higher in AAs than Hispanics and Caucasians. AAs reported more perceived and everyday discrimination than these other racial groups. | | | | | | | | Yes | | | |
| Hahm et al.  (2010) | | Cross-sectional | | | | | | | | | Asian American men (n=972) and women (n=1075) aged 18-75 years, and 16% with HTN, from the National Latino and Asian-American Study (2002-3003) | | | | | | | | | | | Perceived discrimination assessed based on a Detroit area study through self-reported frequency of discrimination | | | | | Respondents were asked if they had ever had or been diagnosed with: Chronic headaches, chronic pain, high blood pressure, and diabetes/high blood sugar | | | | | | | 1 visit, web-based study | | | | | | | | | Level of discrimination was not associated with high blood pressure but was associated with prevalence of diabetes. | | | | | | | | No, diabetes only | | | |
| Krieger et al.  (2010) | | Cross-sectional | | | | | | | | | Non-Hispanic black or AA (n=442) and Caucasian (n=1018) adults aged 25-70 years, and 23% with HTN | | | | | | | | | | | Explicit exposure to racial discrimination assessed using the validated 9-item self-report Experiences of Discrimination instrument.  Implicit measures of exposure to racial discrimination assessed by anchoring language explicitly addressing if the participant had been a target of discriminatory behavior on the Implicit Associations Test. | | | | | Self-reported BP collected on the US National Health Interview Survey | | | | | | | 1 visit | | | | | | | | | Implicit and explicit measures of racial discrimination were not associated with risk of HTN (criteria not reported). | | | | | | | | No | | | |
| McClure et al.  (2010) | | Cross-sectional | | | | | | | | | Latin American adult men (n=46, SBP=118.3±12.0, DBP=72.4±9.0 mmHg) and women (n=86, SBP= 112.7±15.3 mmHg, DBP=73.8±9.3 mmHg) immigrants aged >18 years | | | | | | | | | | | Perceived discrimination and socioeconomic status assessed via self-report and rated by degree of stress based on a 5-point scale | | | | | BP was measured using an oscillometric automatic BP monitor, two times, at least 10 minutes apart | | | | | | | 1 visit | | | | | | | | | Perceived discrimination stress predicted elevated SBP among men but not women. | | | | | | | | Yes, men only | | | |
| Smart et al.  (2010) | | Cross-sectional | | | | | | | | | Black (n=31, SBP=127.7.8±14.7, DBP=75.3±14.7 mmHg) and White (n=31, SBP=123.9±13.6 mmHg, DBP=72.4±7.8 mmHg) adults aged 27±10.2 years | | | | | | | | | | | Perceived discrimination assessed via a 10-item Everyday Discrimination scale pertaining to interpersonal mistreatment over the last 12 months | | | | | BP was measured using ambulatory BP monitors with measures taken three times per hour during the awake periods and once per hour during the sleep period | | | | | | | 1 work day | | | | | | | | | Perceived discrimination was associated with increased SBP and DBP during the day and decreased dipping for HR during the sleeping period. | | | | | | | | Yes | | | |
| Todorova et al.  (2010) | | Cross-sectional | | | | | | | | | Puerto Rican men (n=331) and women (n=791) aged 45-75 years, SBP=135 mmHg, DBP=78.3 mmHg | | | | | | | | | | | Perceived discrimination assessed via 4-item questionnaire | | | | | BP was measured using an automated sphygmomanometer, 6 times throughout the interview, twice at three intervals | | | | | | | 1 visit | | | | | | | | | Perceived discrimination was associated with higher DBP. | | | | | | | | Yes | | | |
| Barksdale et al.  (2009) | | Cross-sectional | | | | | | | | | Black American men (n=64) and women (n=147) aged 25-79 years; SBP= 126.3±21.1 mmHg, DBP=82.9±12.6 mmHg | | | | | | | | | | | Perceived racism was measured using the Likert-type Responses to Perceived Racism Scale | | | | | BP measured 5 minutes after signing consent form and then again 5 minutes after a questionnaire following AHA protocol | | | | | | | 1 visit | | | | | | | | | Racial discrimination was not associated with SBP or DBP. | | | | | | | | No | | | |
| Lewis et al.  (2009) | | Cross-sectional | | | | | | | | | AA (n=2,826) and Caucasian (n=1,868) adults aged 74.1±6.7 from the Chicago Health and Aging Project; SBP=134.6±17.2 mmHg, DBP=77.9±10.9 mmHg | | | | | | | | | | | Perceived discrimination assessed using the Everyday Discrimination scale | | | | | BP measured twice with 30 seconds in between using a sphygmomanometer | | | | | | | 1 visit | | | | | | | | | Perceived discrimination associated with higher levels of DBP in entire sample. Secondary analysis found that perceived discrimination associated with higher levels of DBP in older AAs and not Caucasians. | | | | | | | | Yes | | | |
| Krieger et al.  (2008) | | Cross-sectional | | | | | | | | | Black men (n=308) and women (n=158), Latino men (n=152) and women (n=115), Caucasian men (n=182) and women (n=102), and other ethnicity men (n=73) and women (n=45) union workers aged 24-64 years; SBP=134.8±17.9 mmHg, DBP=78.6±12.9 mmHg | | | | | | | | | | | Self-reported workplace abuse, racial discrimination, and sexual harassment assessed via 45 minute survey | | | | | BP measured 3 times using an automatic sphygmomanometer after 5 minutes of quietly sitting | | | | | | | 1 visit | | | | | | | | | Response to unfair treatment was associated with SBP. | | | | | | | | Yes | | | |
| Rahman et al.  (2008) | | Cross-sectional | | | | | | | | | Predominately black (n=134) men (n=63) and women (n=112), aged 52±13 years, and ~50% with HTN | | | | | | | | | | | Self-reported experiences of racial discrimination assessed using a “well-validated instrument” | | | | | HTN self-reported via face-to-face or mailed survey | | | | | | | 1 visit | | | | | | | | | Experiences of racial discrimination were not associated with self-reported HTN (criteria not reported). | | | | | | | | No | | | |
| Pointer et al.  (2008) | | Cross-sectional | | | | | | | | | Men (n=63) and women (n=113) of self-reported African descent, aged 32±12 years; SBP=121±14 mmHg, DBP=74±11 mmHg | | | | | | | | | | | Chronic exposure to racism assessed using the Perceived Racism Scale | | | | | BP measured using an automatic sphygmomanometer every minute during three resting periods of a 3 hour visit | | | | | | | 1 visit | | | | | | | | | SBP, DBP, and MAP were not associated with perceived racism. | | | | | | | | No | | | |
| Roberts et al.  (2008) | | Cross-sectional | | | | | | | | | AA men (n=393, 57% with HTN) and women (n=717, 55% with HTN) primarily aged >45 years | | | | | | | | | | | Exposure to unfair treatment due to race or other factors were measured using the Everyday Discrimination Scale. | | | | | BP measured using an automatic monitor, taken 3 times, with the 2^nd^ and 3^rd^ readings averaged | | | | | | | 1 visit | | | | | | | | | Women reporting frequent non –racial discrimination had the highest odds of HTN (i.e., SBP > 140 and/or DBP >90 mmHg, and/or on antihypertensive medication) versus those reporting no exposure. | | | | | | | | Yes, women only and in non-racial discrimination | | | |
| Salomon & Jagusztyn  (2008) | | Cross-sectional | | | | | | | | | White (n=28), Black (n=24), and Latino (n=18) college undergraduate men (n=21) and women (n=51) aged 18-30 years, with normal BP | | | | | | | | | | | Perceived discrimination assessed via the Perceived Ethnic Discrimination Questionnaire. Unfair treatment assessed via the Life Experiences Scale | | | | | BP and HR were measured three times using the Accutracker II ambulatory monitor | | | | | | | 1 visit | | | | | | | | | Past discrimination was associated with higher ambulatory SBP among Latinos and lower resting SBP among Caucasians, and associated with attenuated SBP and HR reactivity among Latinos only. | | | | | | | | Yes, Latinos only | | | |
| Singleton et al.  (2008) | | Cross-sectional | | | | | | | | | Black men (n=11) and women (n=41) aged 20-64 years; SBP=118.4±10.2 mmHg, DBP=73.7±7.2 mmHg | | | | | | | | | | | Exposure and coping responses to racism assessed using the Perceived Racism Scale | | | | | BP was measured using 24-hr ambulatory BP monitoring | | | | | | | 1 visit | | | | | | | | | Participants reporting greater use of avoidance in response to perceived racism had higher SBP and DBP. | | | | | | | | Yes | | | |
| Hill et al  (2007) | | Cross-sectional | | | | | | | | | AA men (n=19) women (n=21) college students aged 19.3±1.7 years;  SBP=124.0±10.3 mmHg, DBP=72.6±6.3 mmHg | | | | | | | | | | | Perceived racism assessed using the Perceived Racism Scale | | | | | BP was measured using 24-hr ambulatory BP monitoring | | | | | | | 1 visit | | | | | | | | | Higher perceived frequencies of exposure to racism in academic settings were associated with higher DBP but not in public settings. | | | | | | | | Yes | | | |
| Cozier et al.  (2006) | | Cross-sectional | | | | | | | | | Black women (N=30,330) aged 21 to 69 years, with HTN, from the Black Women’s Health Study | | | | | | | | | | | Perceptions and experiences of racism assessed using the 1997 follow-up questionnaire | | | | | Majority of sample self-reported BP and 115 women participated in a validation visit with BP measured using a standard sphygmonometer | | | | | | | Data from 1 visit | | | | | | | | | Racism was not associated with incident HTN (i.e., SBP > 140 and/or DBP >90 mmHg, and/or on antihypertensive medication) in the total sample, however, positive associations were observed for personally mediated racism in women born outside of the US. | | | | | | | | No, main analysis  Yes, sub-analysis | | | |
| Brown et al.  (2006) | | Cross-sectional | | | | | | | | | AA (n=934; 32% with HTN), Caucasian (n=1549, 17% with HTN), Chinese (n=250, 9% with HTN), Hispanic (n=286, 14% with HTN), and Japanese (n=281; 10% with HTN) women aged approximately 46 years | | | | | | | | | | | Perceived unfair treatment assessed using 10-items from the Everyday Discrimination Scale | | | | | BP was measured using a random-zero sphygmomanometer | | | | | | | 1 visit | | | | | | | | | High levels of perceived unfair treatment were not associated with SBP or DBP. | | | | | | | | No | | | |
| Merritt et al.  (2006) | | RCT | | | | | | | | | Normotensive Black men (N=73) aged 31.7±9.5 years | | | | | | | | | | | Experiment: Randomized to a 2-min audiotaped non-racist or blatantly racist stressor condition followed by preparing a 5-min presentation and completing a 5-min anger recall task regarding the stressor  Manipulation check: Perceived racism assessed using a single question regarding the stressor | | | | | BP and HR measured continuously throughout the experiment with an automatic BP monitor. | | | | | | | 1 visit | | | | | | | | | DBP was higher for the non-racist condition compared to the racist condition during the anger recall task. Participants in the non-racist condition who perceived high levels of racism during active speech showed larger increases in SBP and DBP across post-speech rest, anger recall, and subsequent rest. | | | | | | | | No, main analysis  Yes, sub-analysis | | | |
| Peters  (2006) | | Cross-sectional | | | | | | | | | AA men (n=29) and women (n=133) aged 18-80 years, with no history (n=131), and a history of HTN (n=31) | | | | | | | | | | | Perceived racism assessed using a shorter version of the Racism and Life Experiences Scale and the Krieger Racial Discrimination Questionnaire | | | | | BP was measured twice using automated devices according to JNC guidelines | | | | | | | 1 visit | | | | | | | | | Perceived racism was not associated with SBP or DBP. | | | | | | | | No | | | |
| Ryan et al.  (2006) | | Cross-sectional | | | | | | | | | Black /AA (n=190) and Latinos (n=490) aged >18 years from the New Hampshire Racial and Ethnic Approaches to Community Health 2010 Initiative; SBP=128.7 mmHg, DBP=79.6 mmHg | | | | | | | | | | | Perceived racial/ethnic discrimination assessed using a 10-item survey, adapted from the Reaction to Race module | | | | | BP measured using a digital BP monitor after the survey | | | | | | | 1 visit | | | | | | | | | A U-shaped relationship was found between discrimination and SBP. | | | | | | | | Yes | | | |
| Davis et al.  (2005) | | Cross-sectional | | | | | | | | | AA men (n=160) and women (n=196) with (n=174) and without HTN (n=182) aged 21-81 years from the Metro Atlanta Heart Disease Study | | | | | | | | | | | Perceived racial discrimination assessed via self-report questionnaire | | | | | BP measured in right arm by mercury sphygmomanometer with the last two readings averaged | | | | | | | 1 visit | | | | | | | | | Exposure to racial dissemination was not associated with the prevalence of HTN (i.e., SBP > 140 and/or DBP >90 mmHg, and/or on antihypertensive medication). | | | | | | | | No | | | |
| Din-Dzietham et al.  (2004) | | Cross-sectional | | | | | | | | | AA (n=356) men (n=160) and women (n=196) aged *>*21 years; SBP=129.0±19.8 mmHg, DBP=82.6±12.0 mmHg | | | | | | | | | | | Perceived responses to general stress and racism assessed using a questionnaire adapted from validated study instruments | | | | | BP measured 3 times in right arm by mercury sphygmomanometer with the last two readings averaged | | | | | | | 1 visit | | | | | | | | | The likelihood of HTN increased with higher levels of perceived stress following racism from non-AAs, but not from race-based discrimination from other AAs. The adjusted magnitude that SBP and DBP increased between low and very high levels of stress was greater when race-based discrimination came from AAs compared to non-AAs. | | | | | | | | Yes | | | |
| Peters  (2004) | | Cross-sectional | | | | | | | | | AA men (n=29) and women (n=133) aged 43.6±17.7 years, and 41% with HTN | | | | | | | | | | | Perceived racism assessed using the Racism and Life Experiences Scales short-form and the Krieger Racial Discrimination Questionnaire | | | | | Resting BP measured using automatic BP monitor according to JNC guidelines | | | | | | | 1 visit | | | | | | | | | Perceived racism was not associated with higher SBP, DBP, or BP classification. | | | | | | | | No | | | |
| Clark & Adams  (2004) | | Experimental | | | | | | | | | Black women (N=117) college students aged 26.1±8.83 years; SBP=109.7±13.2 mmHg, DBP=61.5±9.4 mmHg | | | | | | | | | | | Experiment: Ethnicity relevant speaking task used to elicit BP responses                                 Questionnaire: Perceptions of interethnic group racism assessed using the Racism and Life Experiences Scale; Active coping was assessed using The John Henryism Active Coping scale | | | | | BP measured in nondominant arm using portable BP monitor at baseline and at 0.5, 1.5, 2.5, and 3 minutes during task | | | | | | | 1 visit | | | | | | | | | Perceived racism and John Henryism interacted to predict SBP reactivity. | | | | | | | | Yes | | | |
| Clark  (2003) | | Experimental | | | | | | | | | Black men (N=64) college students aged 22.69±6.6 years; SBP=116.7±9.0 mmHg, DBP=65.5±5.3 mmHg | | | | | | | | | | | Experiment: Standardized serial subtraction task to elicit BP response  Questionnaire: Perceived racism assessed using modified Life Experience and Stress Scale, and social support assessed using Sarason Social Support Scale | | | | | BP measured in nondominant arm using portable BP monitor at baseline and at 0.5, 1.5, and 2.5 minutes during task | | | | | | | 1 visit | | | | | | | | | Perceived racism interacted with quantity of social support to predict changes in SBP during a standardized serial subtraction test, but it was not an independent predictor of BP. | | | | | | | | Yes | | | |
| Steffen et al.  (2003) | | Cross-sectional | | | | | | | | | AA men (n=30) and women (n=39) aged 34±6 years from the Duke Biobehavioral Investigation of Hypertension study; SBP=126.0±17.0 mmHg, DBP=82.0±14.0 mmHg | | | | | | | | | | | Perceived racism was assessed using the 51-question Perceived Racism Scale | | | | | Seated BP measured three times, 2 minutes apart, during each visit using a mercury column sphygmomanometer; BP also taken during a typical work day using an ambulatory BP monitor | | | | | | | 3 visits, each 1 week apart for clinic BP | | | | | | | | | Perceived racism was associated with higher ambulatory SBP and DBP during the waking hours. | | | | | | | | Yes | | | |
| Blascovich et al.  (2001) | | RCT | | | | | | | | | AA (n=20) and European-American (n=19) university students; BP values not reported | | | | | | | | | | | Experiment: Randomized to either view a stereotype-threat condition videotape or a low-stereotype-threat condition videotape followed by completing the Remote Associates Test to assess verbal ability | | | | | MAP recorded continuously using an automatic BP monitor. | | | | | | | 1 visit | | | | | | | | | AA under stereotype threat exhibited larger increases in MAP during the Remote Associations Test, and performed more poorly on difficult test items. | | | | | | | | Yes | | | |
| Fang & Myers  (2001) | | Experimental | | | | | | | | | AA (n=31, SBP=115.4±9.4 mmHg, DBP=61.8±6.4 mmHg) and Caucasian (n=31, SBP=114.2±8.8 mmHg, DBP=64.5±6.9 mmHg) undergraduate men aged 19.7 years | | | | | | | | | | | Experiment: Viewed 3 film excerpts that depicted neutral, anger-provoking (but race-neutral), and racist situations  Manipulation check: Emotions assessed following videos using a mood checklist from the Profile of Mood States | | | | | BP measured using an automated BP monitor | | | | | | | 1 visit | | | | | | | | | Participants exhibited significantly greater DBP reactivity to anger-provoking and racist stimuli compared with neutral control stimuli, but no difference was found among racial groups. | | | | | | | | Yes, but no differences by race | | | |
| Guyll et al.  (2001) | | Experimental | | | | | | | | | AA (n=101, SBP=114.9±16.5 mmHg, DBP=73.4±9.5 mmHg) and European American (n=262, SBP=109.2±13.6 mmHg, DBP=70.3±9.1 mmHg) women aged 45.5±2.4 years from the longitudinal SWAN study | | | | | | | | | | | Experiment: Completed a nonsocial stressor mirror tracing task and a social stressor speech task.  Questionnaires: Self-administrated questionnaires to assess experiences of mistreatment and discrimination | | | | | BP and HR measured using an automated BP monitor at baseline and the 0.5 and 2-min marks during each of the reactivity tasks | | | | | | | 1 visit | | | | | | | | | Mistreatment associated with increased DBP among AAs but not European Americans. AAs who attributed mistreatment to racial discrimination exhibited greater average DBP reactivity. | | | | | | | | Yes | | | |
| Clark  (2000) | | Cross-sectional | | | | | | | | | AA graduate and undergraduate women (N=39) aged 24.4±3.79 years, with normal BP | | | | | | | | | | | Experiment: A speech task including 3-minute preparation and 3-minute speech presentation  Questionnaires: Perceptions of racism, psychological, and coping responses to racism assessed via the Perceived Racism Scale | | | | | BP measured using an automatic monitor before, during, and after speaking task | | | | | | | 1 visit | | | | | | | | | Perceived racism was associated with DBP during the speech, early recovery, and late recovery. | | | | | | | | Yes | | | |
| Krieger & Sidney  (1996) | | Cross-sectional | | | | | | | | | Black (n=1,974) and White (n=2,112) men (n=1,837) and women (n=2,249) aged approximately 31 years, enrolled in the CARDIA study, with normal BP | | | | | | | | | | | Racial discrimination and unfair treatment assessed using a previously developed self-administered questionnaire | | | | | BP measured 3 times in one minute intervals by trained and certified technicians using a random zero sphygmomanometer | | | | | | | 1 visit | | | | | | | | | Experiences of racial discrimination were reported by 80% of the AA sample and these experiences associated with BP (SBP vs DBP not specified). | | | | | | | | Yes | | | |
| McNeilly et al.  (1995) | | RCT | | | | | | | | | AA women (N=30) aged 18-33 years, with normal BP | | | | | | | | | | | Individuals randomized (n=15 per group) to participate in racist and non-racist debate stressor, in counterbalance order  Manipulation check: Asked to rate mood on visual analogue scale | | | | | Resting BP measured using automatic BP monitor | | | | | | | 1 visit | | | | | | | | | Participants had greater SBP, DBP, and HR reactivity while verbally responding during racist stressor compared to the non-racist stressor. There was also greater SBP and HR reactivity while listening to the racist stressor compared to the non-racist stressor, and higher SBP and DBP during recovery from the racist stressor compared to the non-racist stressor. | | | | | | | | Yes | | | |
| Armstead et al.  (1989) | | RCT | | | | | | | | | Black men (n=12) and women (n=15) college students aged 21.4±2.6 years; SBP=115.2 mmHg, DBP=71.5 mmHg | | | | | | | | | | | Experiment: Each participant viewed films (1.5 min in length each) consisting of three stimulus conditions – neutral control, racist, and anger provoking in random order  Manipulation check: Asked to rate mood on mood checklist | | | | | BP measured using a sphygmomanometer according to AHA guidelines before and after each film | | | | | | | 1 visit | | | | | | | | | SBP and MAP increased during the presentation of racist stimuli but not of an anger-provoking or neutral stimuli. | | | | | | | | Yes | | | |
| James et al (1984) | | Cross-sectional | | | | | | | | | Black men (N=112, 31% with HTN) aged 17- 60 years | | | | | | | | | | | Perceived racism hindrance to job success assessed with a single question | | | | | BP measured using auscultation method according to Hypertension Detection and Follow-up Program guidelines | | | | | | | 1 visit | | | | | | | | | Job success and SBP as well as DBP were not modified by perceived racism. | | | | | | | | No | | | |
| ***Weight*** | | | | | | | | | | | | | | | | | | | | | | | | | | | | | | | | | | | | | | | | | | | | | | | | | | | | | | |
| Major et al.  (2012) | | | Experimental | | | | | | | | | Women (N=99) aged 18.8±1.3 years who perceived themselves as overweight and had a BMI of 27.4±5.6 | | | | | | | | | | Randomized to either give a video-taped or an audio-taped speech on “why you would make a good dating partner” to evaluators | | | | Continuous recordings of BP were measured during the video-taped or audio-taped speech using an automatic BP monitor; MAP reactivity was calculated | | | | | | | | 1 visit | | | | | | | | | Higher BMI was associated with increased MAP when weight was visible to evaluators and concerns about stigma were activated but not when weight was not visible to evaluators. | | | | | | | | Yes | | | |
| ***Multiple Types of Stigma/Discrimination*** | | | | | | | | | | | | | | | | | | | | | | | | | | | | | | | | | | | | | | | | | | | | | | | | | | | | | | |
| Krieger, N.  (1990) | | | | Cross-sectional | | | | | | | | AA (n=51) and Caucasian (n=50) women aged 20-80 years | | | | | | | | | | Response to unfair treatment and gender and race discrimination assessed via questionnaire administered during a 20-minute telephone interview | | | | | | HTN status determined by asking respondents if they had ever been diagnosed with high blood pressure | | | | | 1 phone interview | | | | | | | | | AA women who internalized unfair treatment and recounted less racist or sexist incidents were at greater risk for high BP (self-reported). There was no association found for Caucasian women. | | | | | | | | | Yes | | | |
| **Heart Rate / Heart Rate Variability as Primary Cardiovascular Health Outcome (n=6)** | | | | | | | | | | | | | | | | | | | | | | | | | | | | | | | | | | | | | | | | | | | | | | | | | | | | | | |
| ***Race*** | | | | | | | | | | | | | | | | | | | | | | | | | | | | | | | | | | | | | | | | | | | | | | | | | | | | | | |
| Hill et al.  (2017) | | | | | Cross-sectional | | | | | | | AA men (n=43) and women (n=56) aged 19.9±2.8 years | | | | | | | | | | Racial discrimination assessed using the Perceived Ethnic Discrimination Questionnaire-Community Version | | | | | | HRV measured via ECG | | | | | | | 1 visit | | | | | | | | | Greater lifetime burden of racial discrimination and discriminatory harassment and/or assault were associated with lower resting high-frequency HRV. | | | | | | | Yes | | | |
| Kemp et al.  (2016) | | | | | Cross-sectional | | | | | | | Self-identified Brown (n=3,502), White (n=6,467), and Black (n=2020) men (n=5,468) and women (n=6,521) aged 35 to 74 years from the ELSA-Brasil Cohort Study | | | | | | | | | | Perceived discrimination assessed using the Everyday Discrimination Scale | | | | | | HRV measured via ECG | | | | | | | 1 visit | | | | | | | | | Although black and brown individuals displayed higher, high-frequency HRV relative to white individuals, high-frequency HRV was partially mediated by racial discrimination. | | | | | | | Yes | | | |
| Hoggard et al.  (2015) | | | | | Experimental | | | | | | | AA women (N=42) college students aged 19.8±2.1 years | | | | | | | | | | Experiment: Women randomized to either an AA or a European American “perpetrator” who led a scripted racial discrimination dialogue in the presence of the women on day one and the women reflected on the racial event on day two  Manipulation checks: Two questions assessing perception of the racial event | | | | | | HRV measured via ECG; HR calculated in beats per minute | | | | | | | 2 days | | | | | | | | | Women insulted by the European American “perpetrator” exhibited lower HRV during day one and higher HR during day two, while women who were insulted by the AA “perpetrator” exhibited an increase in HRV during day one and a slight decline in HRV during day two. HRV frequency not reported. | | | | | | | Yes | | | |
| Wagner et al.  (2013) | | | | | Cross-sectional | | | | | | | Black (n=16) and White (n=16) women aged 53.9±8.9 years with type 2 diabetes | | | | | | | | | | Experiment: Public speaking stressor in which participants defended themselves against false acquisition of shoplifting  Questionnaires: Racial discrimination assessed using the 18-item Schedule of Racist Events; Racial attribution via single question | | | | | | HRV measured via ECG; Cortisol and norepinephrine via serum; BP and HR measured using semi-automatic BP monitor | | | | | | | 1 visit | | | | | | | | | Higher discrimination associated with lower high-frequency HRV during the stressor. There was no association between lifetime discrimination and sympathetic markers. | | | | | | | Yes | | | |
| Utsey et al.  (2007) | | | | | Cross-sectional | | | | | | | AA undergraduate college student men (n=83) and women (n=132) aged 19.4±2.5 years | | | | | | | | | | Lifetime experience of race-related stress assessed using the Index of Race-Related Stress-Brief Version | | | | | | HRV and HR measured via Polar 810s Heart Rate Monitor | | | | | | | 1 visit | | | | | | | | | HRV moderated the relationship between institutional race-related stress and psychological distress for men, but not women. The high and low frequency domains of HRV were not disclosed in the study findings. | | | | | | | Yes, men only | | | |
| ***Weight*** | | | | | | | | | | | | | | | | | | | | | | | | | | | | | | | | | | | | | | | | | | | | | | | | | | | | | | |
| Kube et al.  (2016) | | | | | | Experimental | | | | | | Women with obesity (n=14) and without obesity (n=14) aged 25.3±2.9 years | | | | | | | | | | Experiment: Simplified version of MID task; Adaptation of SID task  Questionnaire: Self-paced computerized face rating; History of negative social experiences using Perception of Teasing Scale | | | | | | HRV measured via ECG; HR was estimated in 500-ms intervals | | | | | | | | 1 visit | | | | | | | | | Women with obesity relative to controls demonstrated diminished heart rate responses to negative social outcomes. Differences in cardiac responses in women with obesity were moderated by weight-related teasing experiences. | | | | | | | Yes | | |
| **Blood & Saliva Cardiovascular Biomarkers (n=18)** | | | | | | | | | | | | | | | | | | | | | | | | | | | | | | | | | | | | | | | | | | | | | | | | | | | | | | |
| ***Race*** | | | | | | | | | | | | | | | | | | | | | | | | | | | | | | | | | | | | | | | | | | | | | | | | | | | | | | |
| Lucas et al.  (2017) | | | | | | | Experimental | | | | | | AA men (n=21) and women (n=64) aged 31.1±13.6 years | | | | | | | Experiment: TSST was used to induce mild psychosocial stress and associated physiological responses  Questionnaires: Perceived racial discrimination assessed using the Everyday Discrimination Scale; Racial identity assessed using the 8-item centrality subscale of the Multidimensional Inventory of Black Identity | | | | | | | | Oral fluids assayed for 4 stress-related salivary analytes including Alpha-amylase, cortisol, DHEA, and C-reactive protein measured at baseline and recovery phases of the stressor | | | | | | | | | 1 visit | | | | | | | | | When racial identity was strong, highly perceived discrimination was associated with low hypothalamic-pituitary-adrenal axis activity at baseline, low stress mobilization during the test, and a robust inflammatory response during recovery | | | | | | Yes | | |
| Lucas et al.  (2016) | | | | | | | Experimental | | | | | | AA men (n=36) and women (n=82) aged 31.6±13.8 years | | | | | | | Experiment: TSST was used to induce mild psychosocial stress and associated physiological responses. Two randomized manipulations implemented during the task and participants given either high or low levels of justice.  Questionnaires: Attributions of racism assessed using 4 questions; Justice beliefs assessed using Procedural and Distributive Justice Beliefs scale  Manipulation check: Administered at beginning of recovery phase of stressor task with questions assessing effectiveness of the distributive justice and the procedural justice manipulations. | | | | | | | | Oral fluids assayed for salivary analytes including cortisol and C-reactive protein measured at baseline and recovery phases of the stressor | | | | | | | | | 1 visit | | | | | | | | | Cortisol and C-reactive protein responses to low distributive justice were higher when procedural justice was low among AAs with a strong belief in justice and perceived racism. | | | | | | Yes | | |
| Giurgescu et al.  (2016) | | | | | | | Cross-sectional | | | | | | AA women (N=96) aged 23.6±5.1 years, during second trimester of pregnancy | | | | | | | Perceived lifetime discrimination assessed using the Experiences of Discrimination | | | | | | | | Systemic inflammation assessed with plasma levels of interleukin-1β, 2, 4, 6, 8, & 10 | | | | | | | | | 1 visit | | | | | | | | | Experiences of racial discrimination were associated with higher cytokine levels of interleukin 4 & 6. | | | | | | Yes | | |
| Brody et al.  (2015) | | | | | | | Longitudinal | | | | | | AA (N=160) aged 17-19 years at enrollment from the Adults in the Making prevention trial | | | | | | | Perceived racial discrimination assessed using nine items from a version of the Schedule of Racist Events | | | | | | | | Serum blood collected to assess interlueken-1β, 6, 8, & 10, and tumor necrosis factor-α and interferon-ᵧat age 22 | | | | | | | | | 3 years | | | | | | | | | Youth exposed to high levels of racial discrimination evinced elevated cytokine levels 3 years later, but was not shown for youth with positive racial identities. | | | | | | Yes | | |
| Zeiders et al.  (2014) | | | | | | | Cross-sectional | | | | | | Caucasian/White (n=76), AA/Black (n=11), Asian (n=8), Hispanic/Latino (n=19), Pacific Islander (n=1), multiethnic /multiracial (n=15), and other (n=10) men (n=38) and women (n=102) aged 22.8±.84 from wave 4 of a larger two-site longitudinal project | | | | | | | Perceived discrimination assessed using the Everyday Discrimination Scale | | | | | | | | Salivary cortisol collected six times per day over three consecutive days: wake-up, 40 min after waking, three semi-random time points at approximately 2, 8, and 12 hr post-awakening, and bedtime | | | | | | | | | 3 days | | | | | | | | | Perceived discrimination predicted flatter diurnal cortisol slopes for racial/ethnic minority individuals only. | | | | | | Yes | | |
| Cunningham et al.  (2012) | | | | | | | Cross-sectional | | | | | | Black (n=1,515) and White (n=1,821) men (n=1,477) and women (n=1,859) aged ~32 years from the CARDIA study | | | | | | | Perceived experiences of racial/ethnic discrimination assessed using the seven-item, situation version of the Experiences of Discrimination index during years 7 and 15 of larger study | | | | | | | | C-reactive protein from blood samples using a Behring Nephelometer II during years 7, 15, and 20 of larger study | | | | | | | | | 20 years | | | | | | | | | Perceived racial/ethnic discrimination was associated with inflammation in both AA and Caucasian groups of women but not AA or Caucasian groups of men | | | | | | Yes, women only | | |
| Lewis et al.  (2010) | | | | | | | Cross-sectional | | | | | | AA men (n=86) and women (n=210) aged 73.1±6.3 years | | | | | | | Daily discrimination was assessed using the Detroit Area Study Everyday Discrimination Scale | | | | | | | | C-reactive protein via plasma blood specimen | | | | | | | | | 1 visit | | | | | | | | | Daily discrimination was associated with higher levels of C-reactive protein. | | | | | | Yes | | |
| Cooper et al.  (2009) | | | | | | | Cross-sectional | | | | | | Black (n=51) and White (n=65) men (n=57) and women (n=59) aged 36.5±7.8 years | | | | | | | Exposure to discrimination was assessed using the Perceived discrimination subscale of the Scale of Ethnic Experience | | | | | | | | Plasma endothelin-1 was sampled upon awakening after an overnight admission | | | | | | | | | 1 visit | | | | | | | | | Increased discrimination associated with increased endothelin-1 among AAs but not Caucasians, regardless of socioeconomic status | | | | | | Yes | | |
| Tull & Chambers  (2001) | | | | | | | Cross-sectional | | | | | | Black men (n=13) and women (n=14) with type 2 diabetes aged 58.7±11.2 years, and Black men (n=24) and women (n=31) controls without type 2 diabetes aged 58.1±10.9 years from the U.S. Virgin Islands | | | | | | | Measurement of internalized racism not specified | | | | | | | | Fasting blood glucose measurement not specified | | | | | | | | | 1 visit | | | | | | | | | Internalized racism was associated with glucose intolerance among AAs with type 2 diabetes. | | | | | | Yes | | |
| ***Weight*** | | | | | | | | | | | | | | | | | | | | | | | | | | | | | | | | | | | | | | | | | | | | | | | | | | | | | | |
| Rodriguez et al.  (2016) | | | | | | | RCT | | | | | | | Men (n=26) and women (n=83) university students aged 19.6±1.7 years | | | | | | | | | Experiment: Randomly assigned to wear “fat suit” or control condition and walked across campus and offered candy and soda at end of walk to activate experiences of weight discrimination  Questionnaires: Anger, anxiety, and depressed mood was assessed using items from the Profile of Mood States anger subscale; Hurt feelings was assessed using the Leary and Springer Hurt Feelings Scale’ Self-esteem assessed using the Four Basic Needs Questionnaire; Antifat attitudes was assessed using the Antifat Attitudes Questionnaire  Manipulation check: Feelings of rejection assessed using subscale from the Four Basic Needs questionnaire | | | | | Salivary cortisol collected via passive drool at baseline and after the eating and drinking tasks. | | | | | | | | | 1 visit | | | | | | | | | Experimentally manipulating apparent body size led participants to consume more unhealthy foods and report higher levels of negative effect, but no differences in cortisol reactivity was found between the experiment and control groups. | | | | | | No, main analysis  Yes, sub-analysis | | |
| Himmelstein et al.  (2015) | | | | | | | RCT | | | | | | | Undergraduate women (N=110) aged 19.8±4.8 years | | | | | | | | | Experiment: Randomized to a experimentally manipulated clothes shopping, weight stigma condition or a control condition  Questionnaires: Self-perceived body weight from a single question; Negative affect assessed using the state of negative affect subscale of the Positive and Negative Affect Schedule  Manipulation check: Tested whether negative affect differed by condition after experiment | | | | | Salivary cortisol collected via passive drool at baseline and 30-minutes post-manipulation | | | | | | | | | 1 visit | | | | | | | | | Participants who perceived themselves as heavy exhibited sustained cortisol elevation post-manipulation compared with individuals in the non-weight stigma control condition. | | | | | | Yes | | |
| Schvey et al.  (2014) | | | | | | | RCT | | | | | | | Lean (n=69) and overweight (n=54) adult women with a mean age ~27 years | | | | | | | | | Experiment: Randomized to watch a 10-min video exposure containing weight-based discrimination or a neutral video  Questionnaires: Positive and negative effect assessed using the Positive Affect Negative Affect scale; Depressive symptoms assessed using the Beck Depression Inventory; Fat phobia assessed using the Fat Phobia Scale; Perceived stress assessed using the Perceived Stress Scale; Emotional reactions to the video assessed using the Post-Video Questionnaire | | | | | Salivary cortisol collected via cotton swab before and 30-minutes after watching video | | | | | | | | | 1 visit | | | | | | | | | Participants in the stigmatizing condition exhibited greater cortisol reactivity compared with those in the neutral condition, irrespective of weight status. | | | | | | Yes | | |
| Sutin et al.  (2014) | | | | | | | Cross-sectional | | | | | | | Overweight or obese (BMI >25) men (n=3,179) and women (n=4,215) from the Health and Retirement Study aged 67.2±9.7 years | | | | | | | | | Perceived discrimination assessment not specified | | | | | High sensitivity C-reactive protein collected via finger prick | | | | | | | | | 1 visit | | | | | | | | | Weight discrimination was associated with higher circulating C-reactive protein, and was moderated by BMI. | | | | | | Yes | | |
| Tomiyama et al.  (2014) | | | | | | | Cross-sectional | | | | | | | Subsample of overweight or obese women (N=47) aged 40.9±7.3 years | | | | | | | | | Exposure of weight stigma assessed using the Stigmatizing Situations Inventory; Consciousness of weight stigma assessed using the Stigma Consciousness Scale | | | | | Salivary cortisol collected via standard diurnal cortisol sampling 4 days at awakening, 30 min post awakening, and across 3 days hourly between 1:00 and 4:00pm, and bedtime; Oxidative stress assessed via 1 fasting blood sample; Adiposity measured via DEXA | | | | | | | | | 4 days | | | | | | | | | Exposure to weight stigma associated with cortisol awakening response and oxidative stress independent of abdominal fat. | | | | | | Yes | | |
| Tsenkova et al.  (2011) | | | | | | | Cross-sectional | | | | | | | Men (n=403) and women (n=535) aged 56.9±11.6 years from the Midlife in the United States survey | | | | | | | | | Perceived daily weight discrimination assessed using nine questions | | | | | Nondiabetic glycemic control (indexed by HbA1c) | | | | | | | | | Data from 1 time point | | | | | | | | | Participants who had higher waist-to-hip ratios and reported weight discrimination had the highest HbA1c levels. | | | | | | Yes | | |
| ***Sexual Orientation*** | | | | | | | | | | | | | | | | | | | | | | | | | | | | | | | | | | | | | | | | | | | | | | | | | | | | | | |
| Doyle & Molix  (2016) | | | | | | | Cross-sectional | | | | | | | | Gay men (n=78) and Lesbian women (n=21) aged 34.6±13.0 years | | | | | | | | Perceived discrimination assessed using two items from the Everyday Discrimination Scale | | | | | | Interleukin-6 measured via saliva sample | | | | | | | | | 1 visit | | | | | | | | Perceived discrimination was predictive of higher levels of interleukin-6 for gay men engaged in less covering but not for those who engaged in more covering. Greater perceived discrimination was associated with lower levels of IL-6 among Lesbian women. | | | | | | Yes, gay men only | | |
| Hatzenbuehler & McLaughlin  (2014) | | | | | | | Cross-sectional | | | | | | | | Lesbian/gay (n=42) and bisexual (n=32) men (n=34) and women (n=40) aged 23.7±4.1 years | | | | | | | | Experiment: Participants exposed to a laboratory stressor and completed the Trier Social Stress Test, a social-evaluative threat task  Questionnaire: Stigma assessed using a 6-item Perceived-Devaluation Discrimination Scale | | | | | | Salivary cortisol collected via drool method at baseline and 20 minutes after each (two) Trier Social Stress Test task | | | | | | | | | 1 visit | | | | | | | | Lesbian, gay, and bisexual young adults who were raised in highly stigmatizing environments as adolescents had a blunted cortisol response following the Trier Social Stress Test compared to those form low-stigma environments. | | | | | | Yes | | |
| ***Multiple Types of Stigma/Discrimination*** | | | | | | | | | | | | | | | | | | | | | | | | | | | | | | | | | | | | | | | | | | | | | | | | | | | | | | |
| Reynolds et al.  (2015) | | | | | | | Cross-sectional | | | | | | | | | AA (n=399) and Other (n=203) men (n=369) and women (n=233) aged 61.5±10.9 with type 2 diabetes | | | | | | | Perceived race/ethnic, level of education, sex/gender, and language discrimination assessed using items previously validated by the Diabetes Study of North California | | | | | | Glycemic control was assessed via HbA1c value from the electronic medical record using values within the past 6 months | | | | | | | | | Data from 1 time point | | | | | | | | Education discrimination associated with glycemic control. Race, gender, and language discrimination were not associated with poor glycemic control. | | | | | | No, race, gender, language  Yes, education Ed | | |
| **Other Indicators of Cardiovascular Health (n=15)** | | | | | | | | | | | | | | | | | | | | | | | | | | | | | | | | | | | | | | | | | | | | | | | | | | | | | | |
| ***Race*** | | | | | | | | | | | | | | | | | | | | | | | | | | | | | | | | | | | | | | | | | | | | | | | | | | | | | | |
| Everson-Rose et al.  (2015) | | | | | | | | Longitudinal | | | | | | | | | White (39%), Black (26.4%), Chinese (12.2%), and Hispanic (22.3%) men (n=3,072) and women (n=3,436) aged 62.0±10.2 years from MESA | | | | | | Perceived lifetime discrimination assessed using the Lifetime Discrimination Scale; Perceived everyday discrimination assessed using the Everyday Discrimination Scale | | | | | | | Cardiovascular events included incident myocardial infarction, resuscitated cardiac arrest, coronary revascularization, definite angina, fatal or nonfatal stroke, and death due to CVD, as defined by the MESA protocol. | | | | | | | | | 10.1 year follow-up | | | | | | | | Participants who experienced lifetime racial discrimination in >2 domains had a 38% greater risk of incident CVD than those reporting no lifetime discrimination. Everyday discrimination was associated with incident CVD in men only. | | | | | | Yes | |
| Neblett et al.  (2013) | | | | | | | | Cross-sectional | | | | | | | | | AA men (n=45) and women (n=60) college students aged 20.7±1.8 years | | | | | | Race-related beliefs and attitudes assessed using the Multidimensional Model of Racial Identity; Experimental session with racism analogues. | | | | | | | Respiratory sinus arrhythmia via spectral analysis; Cardiac pre-ejection period via onset of ECG; HRV measured via ECG and impedance cardiography signals | | | | | | | | | 1 visit | | | | | | | | Personal significance of race as well as personal feelings about AAs and feelings about how others view AAs moderated autonomic responses to the vignettes. | | | | | | Yes | |
| Wagner et al.  (2013) | | | | | | | | Cross-sectional | | | | | | | | | White (n=94) and minority (n=19) women with (n=49) and without (n=64) diabetes with a mean age ~60 years | | | | | | Experiment: Five min of mental arithmetic with harassment  Questionnaires: Perceived lifetime discrimination was assessed using the 9-item Experiences of Discrimination scale; Perceived stress assessed using the Perceived Stress Scale; Stressful life events unrelated to discrimination were controlled using the Social Readjustment Rating Scale | | | | | | | Flow-mediated endothelial function assessed according to international guidelines; Peak HR and peak BP measured via semi-automatic digital manometer; Degree of vasoconstriction via ultrasound  All measurements taken before and after experiment | | | | | | | | | 1 visit | | | | | | | | Perceived lifetime racial discrimination was associated with attenuated flow-mediated dilation at recovery. Neither race/ethnicity nor diabetes status moderated the effect. | | | | | | Yes | |
| Chae et al.  (2012) | | | | | | | | Cross-sectional | | | | | | | | | Black American men (n=1,847) and women (n=3,175) aged 41.9±0.5 years from NASL | | | | | | Racial discrimination assessed using the Major Experiences of Discrimination; Mood disorder assessed using a modified World Health Organization Composite International Diagnostic Interview | | | | | | | History of CVD assessed through self-report | | | | | | | | | Data from 1 time point | | | | | | | | Participants with a history of mood disorder who reported high levels of racial discrimination had the greatest risk of CVD | | | | | | Yes | |
| Mwendwa et al.  (2011) | | | | | | | | Cross-sectional | | | | | | | | | AA women (N=110) aged 47.0±10.9 years from the Minority Organ Tissue Transplant Education Program Stress and Psychoneuroimmun-ological Factors in Renal Health and Disease Study | | | | | | Perceived racism assessed using the Perceived Racism Scale; Perceived stress assessed using the Perceived Stress Scale | | | | | | | Weight and height measured using a balance scale. Height and weight were used to calculate BMI. | | | | | | | | | 1 visit | | | | | | | | BMI was associated with behavioral coping responses to perceived racism. Obese AA women used more behavioral coping responses to perceived racism compared to normal-weight and overweight AA women. | | | | | | Yes | |
| Peek et al.  (2011) | | | | | | | | Cross-sectional | | | | | | | | | Non-Hispanic White (n=1,591), AA (n=416), Hispanic (n=87), Multiracial (n=49), and Other (n=95) men (n=1,132) and women (n=1,106) aged 59.4 years from the Behavioral Risk Factor Surveillance System | | | | | | Self-reported discrimination in healthcare assessed using a single question: “Within the past 12 months, when seeking healthcare, do you feel your experiences were worse than other races, the same as other races, better than other races, or worse than some but better than others?” “Worse than other races” was considered discrimination | | | | | | | Three classes of diabetes outcomes including diabetes quality of care, diabetes self-management, and diabetes complications | | | | | | | | | Data from 1 time point | | | | | | | | Self-reported healthcare discrimination was associated with measures of quality of care, HbA1c testing, and earlier eye examination interval and health outcomes. | | | | | | Yes | |
| Cardarelli et al.  (2010) | | | | | | | | Cross-sectional | | | | | | | | | Non-Hispanic White (n=142), AA (n=167), Hispanic (n=193), with a mean age ~56 years from the North Texas Healthy Heart study, asymptomatic of CHD | | | | | | Perceived racial discrimination and response to unfair treatment was assessed using the Experience of Discrimination instrument | | | | | | | CAC was measured using a 16-slice MSCT scan | | | | | | | | | 1 visit | | | | | | | | Among those who passively responded to unfair treatment, the odds of having CAC present were approximately 3 times higher for those experiencing discrimination. | | | | | | Yes | |
| Thomas et al.  (2006) | | | | | | | | Cross-sectional | | | | | | | | | White (n=76) and Black (n=46) men (n=65) and women (n=57) with a mean age ~37 years | | | | | | Experiences of ethnicity was assessed using the “perceived discrimination” subscale of the Scale of Ethnic Experience | | | | | | | Pressor Responses to Phenylephrine was assessed using ECG signals | | | | | | | | | 1 visit | | | | | | | | Black patients had greater vascular reactivity to phenylephrine than Caucasians. Individuals who perceived more discrimination had a larger increase in DBP in response to phenylephrine. | | | | | | Yes | |
| Troxel et al.  (2003) | | | | | | | | Cross-sectional | | | | | | | | | AA (n=109) and Caucasian (n=225) women aged 46.2±2.5 years | | | | | | Racial discrimination assessed with 10 unfair treatment items on various questionnaires | | | | | | | Carotid ultrasound measurements were assessed using a scanner with linear array-imaging transducer | | | | | | | | | 1 visit | | | | | | | | Among AAs only, the composite stress index and unfair treatment were associated with higher intima-media thickness. AAs who reported experiencing racial discrimination had marginally more carotid plaque than did those who did not report experience racial discrimination. | | | | | | Yes | |
| ***Weight*** | | | | | | | | | | | | | | | | | | | | | | | | | | | | | | | | | | | | | | | | | | | | | | | | | | | | | | |
| Puhl et al.  (2017) | | | | | | | | Longitudinal | | | | | | | | | Underweight, normal weight, overweight, and obese, men (n=788) and women (n=1,042) aged 14.9±1.7 years at baseline and 31.0±1.7 years at follow-up from the Project EAT-IV study | | | | | | | Weight-based teasing by peers assessed by asking participants “Have you ever been teased or made fun of by other kids because of your weight?” Weight-based teasing by family members assessed by asking participants “Have you been teased or made fun of by a family member because of your weight?” | | | | | | Changes in BMI via self-reported height and weight and unhealthy weight control assessed by asking participants “Have you done any of the following things in order to lose weight or keep from gaining weight during the past year?” (Yes/No) | | | | | | | | | Data from 2 time points over 15 years | | | | | | | | | For women, both peer and family-based teasing predicted both increased BMI and unhealthy weight control, and for men, only peer-based teasing predicted higher BMI at follow-up. | | | | | Yes, with differences across gender and teasing source | |
| Jackson et al.  (2014) | | | | | | | | Longitudinal | | | | | | | | | Normal, overweight, and obese, men (n=1,216) and women (n=1,728) aged >50 years, from the English Longitudinal Study of Ageing | | | | | | | Perceived weight discrimination assessed based on items developed and used in the MISUS and the Health and Retirement Study | | | | | | Changes in weight and waist circumference objectively measured | | | | | | | | | Data from 2 time points over 5 years | | | | | | | | | Perceived weight discrimination was associated with increases in weight and waist circumference. An association was also found with odds of becoming obese, but odds of remaining obese did not differ according to experiences of weight discrimination. | | | | | Yes | |
| Sutin & Terracciano  (2013) | | | | | | | | Longitudinal | | | | | | | | | Obese and non-obese men (n=2,549) and women (n=3,608) aged 66.5±10.0 years from the Health and Retirement Study | | | | | | | Perceived everyday weight discrimination assessed using questionnaires from the Health and Retirement Study | | | | | | Changes in weight and waist circumference objectively measured | | | | | | | | | 4 years | | | | | | | | | Participants who experienced weight discrimination were approximately 2.5 times more likely to become obese by follow-up and participants who were obese at baseline were three times more likely to remain obese at follow-up than those who had not experienced discrimination. | | | | | Yes | |
| ***Multiple Types of Stigma/Discrimination*** | | | | | | | | | | | | | | | | | | | | | | | | | | | | | | | | | | | | | | | | | | | | | | | | | | | | | | |
| Udo & Grilo  (2017) | | | | | | | | | Longitudinal | | | | | | | | | Adult men (n=12,011), and women (n=14,981) aged 49.2±16.4 years from the 2001-2002 and 2004-2005 NESARC | | | | | | Perceived experiences with discrimination due to weight, race/ethnicity, and gender assessed based on multiple questions based on the Experiences with Discrimination scales | | | | | | | Cardiovascular conditions assessed via self-reported atherosclerosis, HTN, myocardial infarction, and all other heart diseases; BMI calculated via self-reported height and weight | | | | | | | | | Data from 2 time points over 3 years | | | | | | | | | Perceived weight and racial discrimination were associated with greater likelihood of reporting myocardial infarction and minor heart conditions. Perceived racial discrimination was also associated with greater likelihood of reporting atherosclerosis. | | | | | Yes |
| Clark & Hill  (2009) | | | | | | | | | RCT | | | | | | | | | Normal, overweight, and obese AA men (n=15) and women (n=33) college students, with a mean age of 19 years | | | | | | Experiment: A videotaped scene depicting racism and a neutral scene to examine the effects of body mass on cardiovascular reactivity to racism | | | | | | | A Pulsewave CR 2000 cardiovascular profiling instrument was used to noninvasively assess cardiac output, stroke volume, HR, and BP while viewing the videotaped scenes | | | | | | | | | 1 visit | | | | | | | | | Obese participants had greater stroke volume and cardiac output than participants of normal weight, indicating that obese participants were less emotionally aroused by the stressor. Obese women had the largest drop in heart rate, while obese men had the smallest drop from the stressor period to recovery. | | | | | No |
| Lewis et al.  (2006) | | | | | | | | | Cross-sectional | | | | | | | | | AA women (N=181) aged 50.2±2.8 from the longitudinal SWAN study. | | | | | | Perceived race, ethnicity, age, income level, language, physical appearance, sexual orientation, and other types of discrimination assessed using a modified version of the Detroit Area Study Everyday Discrimination Scale | | | | | | | CAC was quantified via electron beam tomographic scans; Framingham Risk Score was calculated using standard techniques | | | | | | | | | Data averaged over 5 years | | | | | | | | | Chronic exposure to discrimination was associated with CAC, CVD risk factors, and BMI. Recent discrimination was marginally associated with the presence of CAC. Persistent exposure to racial/ethnic discrimination was largely associated with CAC. | | | | | Yes |
|  | | | | | | | | | |  | | | | | | | | |  | | | | | |  | | | | | | |  | | | | | | | | |  | | | | | | | | |  | |  | | |
| *Only findings for primary outcomes are presented, unless a finding for a secondary outcome suggests a significant association. When a measurement outcome is listed in columns 4 and 5 (measurements of discrimination and cardiovascular health indices), but not mentioned in the findings, this indicates that there was not a significant finding for this measurement outcome. Abbreviations: AA=African American; AHA=American Heart Association; BMI=body mass index; BP=blood pressure; CAC=coronary artery calcification; CARDIA=Coronary Artery Risk Development in Young Adults study; CHD=coronary heart disease; CVD=cardiovascular disease; DBP = diastolic blood pressure; DEXA=dual-energy x-ray absorptiometry; DHEA=Dehydroepiandrosterone-sulfate; DODARS=Dominica Obesity and Diabetes Risk Survey; EAT-IV (Eating and Activity in Teens and Young Adults); ECG=electrocardiogram; ELSA=The Brazilian Longitudinal Study of Adult Health; FNS=Fourth National Survey of Ethnic Minorities; HOMA= homeostasis model assessment; HR=heart rate; HRV=heart rate variability; HTN=hypertension; JNC=Joint National Committee; MAP=mean arterial pressure; MESA=Multi-Ethnic Study of Atherosclerosis) MID=monetary incentive delay; Multi-MESA=Ethnic Study of Atherosclerosis; NESARC= National Epidemiologic Survey of Alcohol and Related Conditions; NSAL=National Survey of American Life; NZHS=New Zealand Health; RCT=randomized controlled trial; SBP = systolic blood pressure; SID=social incentive delay; Survey; SWAN=Study of Women’s Health Across the Nation; TSST=Trier Social Stress Test; WC=waist circumference | | | | | | | | | | | | | | | | | | | | | | | | | | | | | | | | | | | | | | | | | | | | | | | | | | | | | | |
